# Construction and validation of an immunoediting-based optimized neoantigen load (ioTNL) model to predict the response and prognosis of immune checkpoint therapy in various cancers

**DOI:** 10.18632/aging.204101

**Published:** 2022-05-25

**Authors:** Xiaofan Su, Haoxuan Jin, Jiaqian Wang, Huiping Lu, Tiantian Gu, Zhibo Gao, Manxiang Li

**Affiliations:** 1 Department of Respiratory and Critical Care Medicine, The First Affiliated Hospital of Xi'an Jiaotong University, Xi'an, Shanxi 710061, China; 2 YuceNeo Technology Co., Ltd., Shenzhen 518000, China; 3 YuceBio Technology Co., Ltd., Shenzhen 518020, China

**Keywords:** neoantigen, immunoediting, checkpoint inhibitors, response, prognosis

## Abstract

Background: Only a minority of patients clinically benefit from immune checkpoint therapy. Tumor clones with neoantigens have immunogenicity; therefore, they are eliminated by T-cell-mediated immune editing. Identifying neoantigen clones with the ability to induce immune elimination may better predict the clinical outcome of immunotherapy.

Methods: We developed ioTNL model, which indicates the immunoediting-based optimized tumor neoantigen load, by identifying tumor clones that could induce immune elimination. Data of more than two hundred patients from our patient pool and previously reported studies who underwent anti-PD-(L)1 therapy were collected to validate the prediction performance of ioTNL model. Clonal architectures, immune editing scores and ioTNL scores were identified. The association between the response as well as prognosis and the ioTNL were evaluated. Panel sequencing of genes from 2,469 patients within 20 cancer types was performed to profile the landscape of immunoediting.

Results: As expected, the ioTNL score could predict the response in patients who underwent immune checkpoint inhibitor (ICI) immunotherapy for various cancers, including non-small cell lung cancer (NSCLC; *p* = 0.0066), skin cutaneous melanoma (SKCM; *p* = 0.026) and nasopharyngeal carcinoma (NPC; *p* = 0.0025). Patients with a high ioTNL score demonstrated longer survival than those with a low score. We verified the ioTNL on our cohort through panel sequencing and found that the ioTNL was associated with the response (*p* = 0.025) and prognosis (*p* = 0.00082) in anti-PD-(L)1 monotherapy. In addition, we found that the immune editing score correlated with the tumor mutation burden (TMB) and the objective response rate of immunotherapy.

Conclusions: Identifying neoantigen clones with the ability to induce immune elimination would better predict the efficacy of immunotherapy. We have proved that the reliable method of ioTNL can be applied to whole-exome sequencing (WES) and panel data and would have a broad application in precision diagnosis in immunotherapy.

## INTRODUCTION

Although immunotherapy using checkpoint inhibitors has significantly improved the overall survival of patients in many malignancies, only a minority of patients achieve benefits [1]. The challenge in predicting the efficacy of checkpoint inhibitors using biomarkers is far from being solved. Checkpoint inhibitors induce tumor elimination by exploiting T cell responses to tumor antigens. Neoantigens, a type of tumor-specific antigens derived from non-silent mutations, are presented by major histocompatibility complex (MHC) molecules and then recognised by T-cell receptors (TCR) as “non-self” peptides.

Several recent studies have revealed that neoantigens are an important factor in determining the response to checkpoint inhibitors [2, 3]. The neoantigen load, which is the sum of putative neoantigens, has been identified as a predictive biomarker in patients treated with checkpoint inhibitors in several clinical cohorts. A disadvantage of assessing the traditional neoantigen load is that different neoantigens are treated equally, although their characteristics are complicated in tumors. Neoantigens derived from truncal mutations were reported to have higher immunogenicity and correlated more with the response than those from non-truncal mutations [2]. The heterogeneity of neoantigens might influence immune surveillance, thereby mediating tumor evolution [4]. A fitness model constructed based on the evolutionary theory of neoantigens proved its ability to predict the efficacy of immunotherapy [3]. For each tumor clone, it is believed that a small set of high-quality neoantigens with high binding affinity to the MHC and TCR, rather than all the putative neoantigens, is sufficient to induce a T-cell response.

A recent study reported that neoantigens could direct immune escape through multiple immunoediting mechanisms under immune selection pressure [2]. Reduced numbers of neoantigens were observed due to a decrease of neoantigen expression or deletion of chromosomal regions containing truncal alterations, thus resulting in immune escape [5, 6]. In some cases, the tumor might lose the heterozygosity of HLA genes or decrease the expression of genes in the neoantigen presentation pathway as an immune escape mechanism [7, 8]. These reports lead to a hypothesis that only the tumor clones with immune elimination capability can be recognised and eliminated by T cells, while immune-edited tumor clones would acquire immune escape from administration. Therefore, patients with a high neoantigen concentration in clones that show immune elimination capability might respond better to immunotherapy.

In this study, we developed an ioTNL score for quantitative characterisation of the neoantigen concentration with immune elimination capability at the patient level to predict the clinical outcome of immunotherapy. We included five cohorts treated with checkpoint inhibitors to investigate the relationship between the ioTNL and patient response or survival (Supplementary Table 1). Our work proved that the ioTNL could predict the clinical outcome following treatment with checkpoint inhibitors. Immunoediting is a relevant predictor of neoantigen immunogenicity that can be considered while selecting neoantigen targets for adoptive cell transfer and vaccine studies.

## RESULTS

### Constructing the neoantigen quantitative model with immune elimination capability

**Figure 1:**
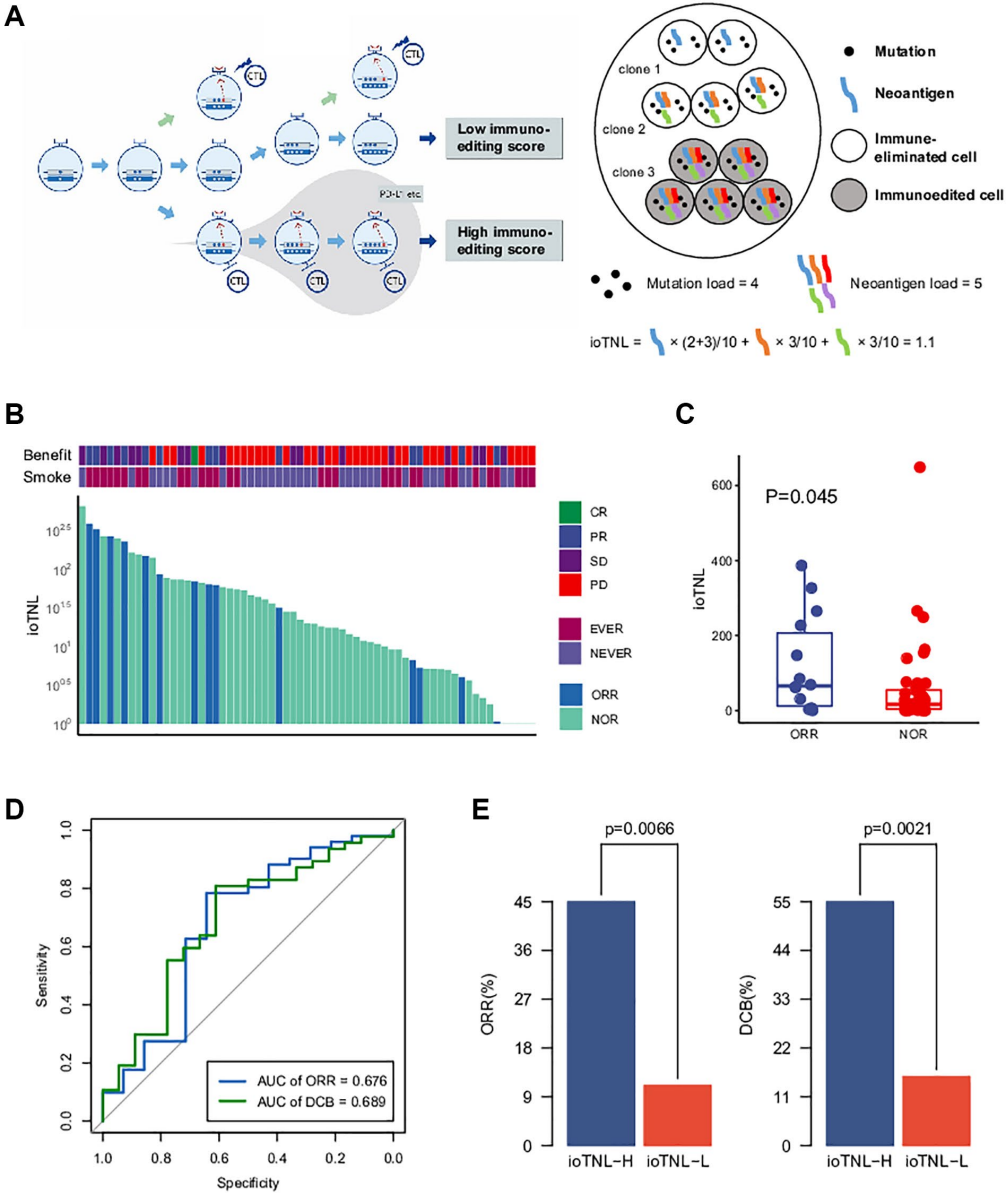
**The model of immunoediting-based optimized neoantigen load (ioTNL).** (**A**) Illustration showing the concept of ioTNL model. Left, due to the instability of the genome, the tumor produces different clones. If tumor clones acquired immune escape and immune tolerance to neoantigens, they would not contribute to the tumor immunogenicity and would be discarded. Only the tumor clones with immune clearance ability would be retained. Right, we demonstrated a hypothetical tumor of three clones. Tumor cells from clone 1 and clone 2 were immune-eliminated cells while tumor cells from clone 3 were immunoedited cells. We assumed that this tumor had five neoantigens so that the tumor neoantigen load was equal to 5. However, neoantigens from immunoedited cells were excluded so that the ioTNL score of this tumor was equal to 1.1. (**B**) Distribution of ioTNL score in the NSCLC cohort. Patients with objective response (ORR) were labeled in blue while patients with non-objective response (NOR) were labeled in cyan. The scores of ioTNL were transformed into log10 format. (**C**) Boxplot of the distribution of ioTNL scores between patients with ORR and NOR. (**D**) Sensitivity and specificity of ioTNL in predicting ORR and DCB in NSCLC cohort. (**E**) Barplots of ORR rate (left) and DCB rate (right) between ioTNL-H group and ioTNL-L group.

We developed ioTNL, an algorithm model that evaluates the concentration of neoantigens in tumor clones with immune elimination capability (Supplementary Figure 1). If a tumor clone acquired immune escape and tolerance to immunoediting neoantigens, it would not contribute to the tumor immunogenicity and would be discarded while calculating the neoantigen load. Only the tumor clones with immune clearance ability would be retained (Figure 1A). The calculation of the ioTNL comprises several steps. First, the cluster of each tumor clone is identified based on the allele frequency and corresponding ploidy of the detected mutations. Second, the immune editing score of each clone is calculated. The tumor was heterogeneous with different clones, and the immune stages of clones were various. We used the ratio of the neoantigen to non-silent mutation to calculate the immune editing score. A tumor clone with a high immune editing score was considered to be in the immune escape stage, otherwise, in the immune elimination stage. Subsequently, the neoantigen load indexes of the immune elimination clones were estimated. Neoantigens are identified as 8 to 11 amino acids for class I MHC-binding peptides, which arise from non-silent mutations. A previous study reported that insertions and deletions (InDels) generate a higher number of neoantigens than missense mutations. Based on the hypothesis that InDel mutations may be more immunogenic than missense mutations, neoantigens derived from InDels were also included in the ioTNL model [9]. Therefore, the design of the ioTNL model is consistent with the idea that truncal neoantigens in clonal have higher immunogenicity and correlated more with the response than subclonal neoantigens [2]. Finally, the sum of the cancer cell fraction of each neoantigen in the immune elimination clones was calculated as the ioTNL score.

### Better response to checkpoint inhibitors is associated with higher ioTNL in multiple cancers

To investigate the implications of the ioTNL in the response to checkpoint inhibitor treatment, we considered a non-small cell lung cancer (NSCLC) cohort from the study by Fang et al. [10]. The cohort included 65 patients who were treated with second-line anti-PD-1/PD-L1 therapy. Previous studies revealed that the tumor mutation burden (TMB) was associated by whole-exome sequencing (WES), and the ioTNL score of each patient was calculated (Supplementary Table 2, Figure 1B). We found that patients with an objective response rate (ORR; [CR/PR]) had significantly higher ioTNL than those with a no objective response (NOR, SD/PD; *p* = 0.045, Figure 1C), as well as in a durable clinical benefit (DCB, *p* = 0.019, Supplementary Figure 2A). The ROC curve showed that the AUCs were 0.676 and 0.689 in predicting the ORR and DCB, respectively (Figure 1D). Patients were divided into ioTNL high and low groups based on the Youden index (the maximum sensitivity and the best specificity under the AUC curve). Patients with high ioTNL had a higher ORR (45% vs. 11%, *p* = 0.007) and DCB (55% vs. 15.6%, *p* = 0.002) than those with low ioTNL (Figure 1E).

**Figure 2:**
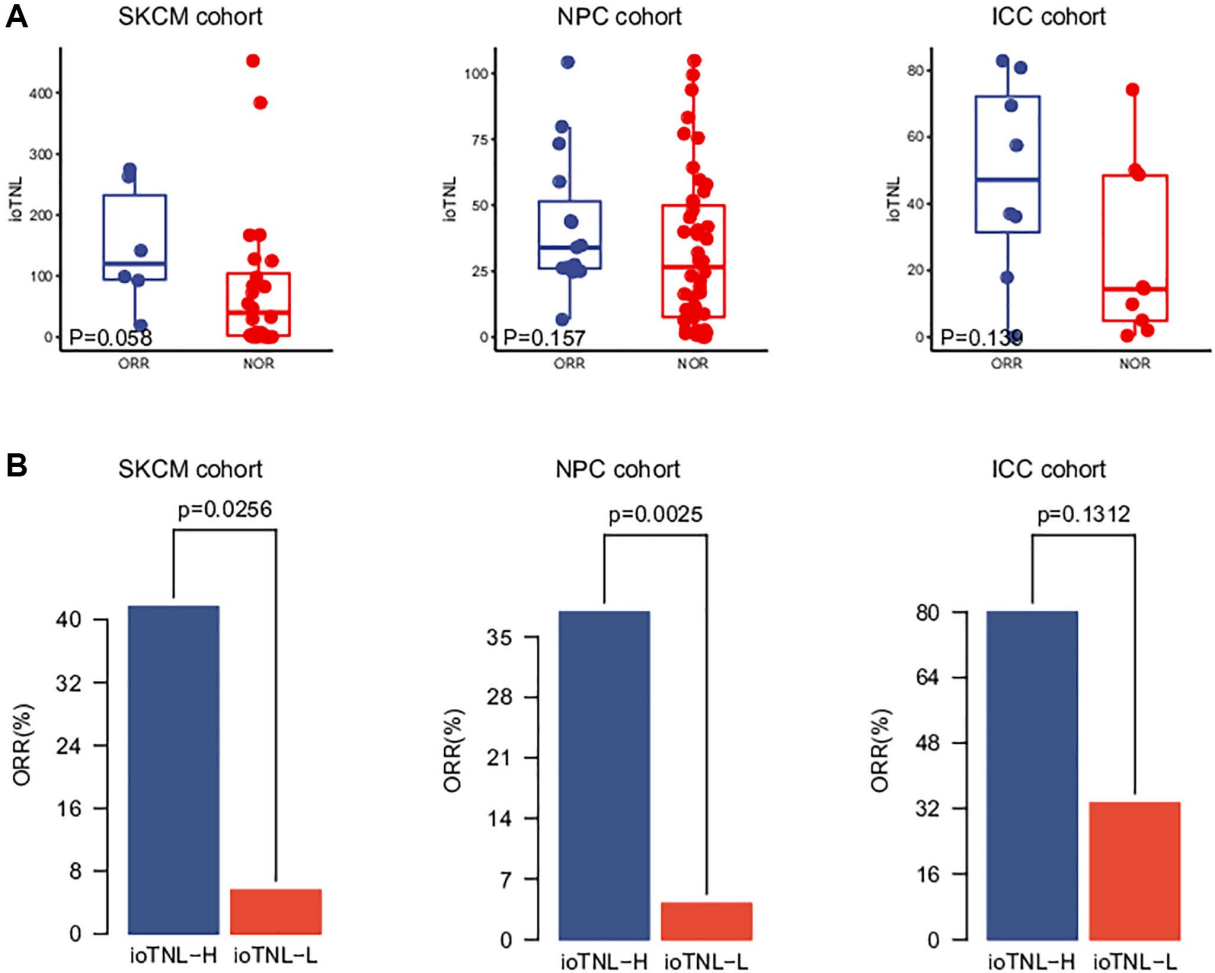
**Validation of ioTNL in multiple datasets.** (**A**) Boxplots of the distribution of ioTNL scores between patients with ORR and NOR in the SKCM cohort (left), the NPC cohort (middle) and the ICC cohort (right). (**B**) Barplots of ORR rate between ioTNL-H group and ioTNL-L group in the SKCM cohort (left), the NPC cohort (middle) and the ICC cohort (right).

We analysed three more clinical cohorts of different cancer types treated with immune checkpoint inhibitors (ICIs) to further validate the prediction efficacy of the ioTNL. The three cohorts were a skin cutaneous melanoma (SKCM) cohort of 64 patients who underwent nivolumab treatment (Riaz et al.), [11] an intrahepatic cholangiocarcinoma (ICC) cohort of 17 patients (Feng et al.) [12] and a nasopharyngeal carcinoma (NPC) cohort of 61 patients (Fang et al.) [13] The ioTNL scores of 33 patients who received nivolumab as the second-line therapy were evaluated in the SKCM cohort (Supplementary Table 3). A relatively higher median ioTNL score was observed in patients who achieved an ORR (*p* = 0.058, Figure 2A, left). Moreover, patients in the ioTNL-high group, determined by the Youden index described above, had a significant ORR (41.7% vs. 5.6%, *p* = 0.0256, Figure 2B, left) compared with those in the ioTNL-low group. A similar result was achieved in the second validation cohort of 61 patients with advanced NPC who received anti-PD-1 therapy alone or in combination with chemotherapy. In contrast to most anti-PD-1-responsive solid tumors, NPC has a modest mutation burden (Supplementary Table 4). Patients with a high ioTNL had a significantly better ORR (*p* = 0.0025, Figure 2B, middle) and DCB (*p* = 0.008, Supplementary Figure 2B). Furthermore, we collected the data of 17 patients with ICC who received the combination of nivolumab and chemotherapy (Supplementary Table 5). Patients who achieved CR or PR showed a tendency of higher ioTNL than those who did not (*p* = 0.139, Figure 2A, right); however, the tendency was not significant due to the small cohort size. In addition, patients in the ioTNL-high group demonstrated a higher ORR than those in the ioTNL-low group (80% vs. 33.3%, *p* = 0.131, Figure 2B, right).

### Higher ioTNL predicts a more favourable prognosis in multiple cancers

**Figure 3:**
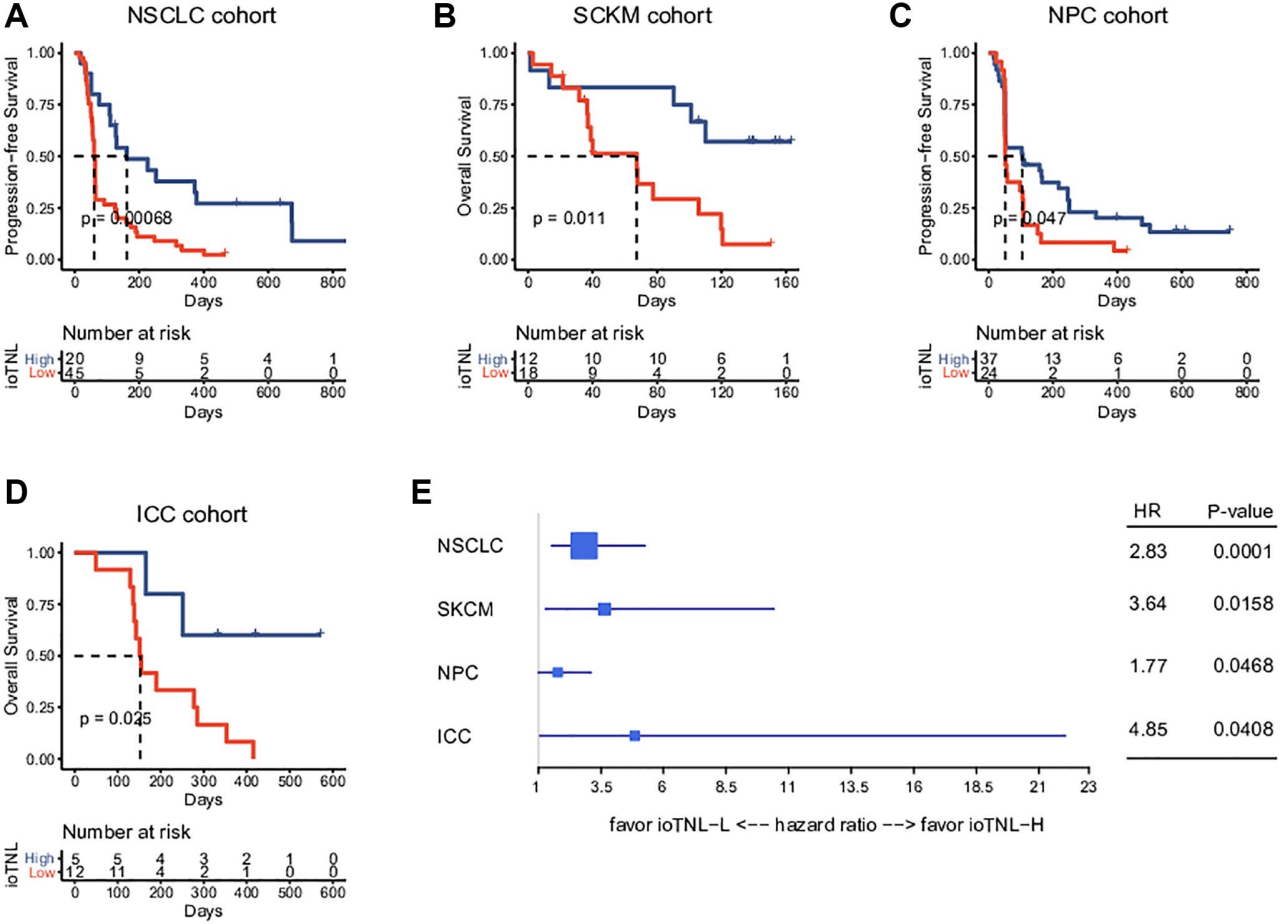
**Prognosis analysis of ioTNL in multiple datasets.** Kaplan-Meier estimator was used to visualize the survival of ioTNL-H group and ioTNL-L group in the NSCLC cohort (**A**), the SKCM cohort (**B**), the NPC cohort (**C**) and the ICC cohort (**D**). (**E**) Forest plot for the hazard ratios of ioTNL in four cohorts. Larger boxes indicate statistical significance.

We examined the association between the ioTNL and prognosis in the NSCLC, SKCM, NPC and ICC cohorts to understand the implications of immunoediting heterogeneity on patient survival. We speculated that patients with a low immune editing score, the neoantigens in which were considered to be in the immune elimination stage, are more likely to benefit from checkpoint inhibitor therapy. As expected, we observed that higher ioTNL scores had a significantly positive association with better survival in these patients. In the NSCLC cohort, progression-free survival (PFS) in patients with a high ioTNL was longer than in those with a low ioTNL (median 161 days vs. 61 days, *P* < 0.001, Figure 3A). In the melanoma cohort, the overall survival (OS) was prolonged (*p* = 0.011, Figure 3B) in patients with a high ioTNL than in those with a low ioTNL. Analysis of the NPC cohort also indicated that the OS was significantly longer in the ioTNL-high than in the ioTNL-low group (median 105 days vs. 52.5 days, *p* = 0.047, Figure 3C). Analysis of the ICC cohort revealed a longer PFS among patients in the ioTNL-high group (*p* = 0.025, Figure 3D). The hazard ratios of above four datasets revealed high ioTNL level was a positive factor for prognosis (Figure 3E).

### Association between the ioTNL score and other immunotherapy biomarkers

**Figure 4:**
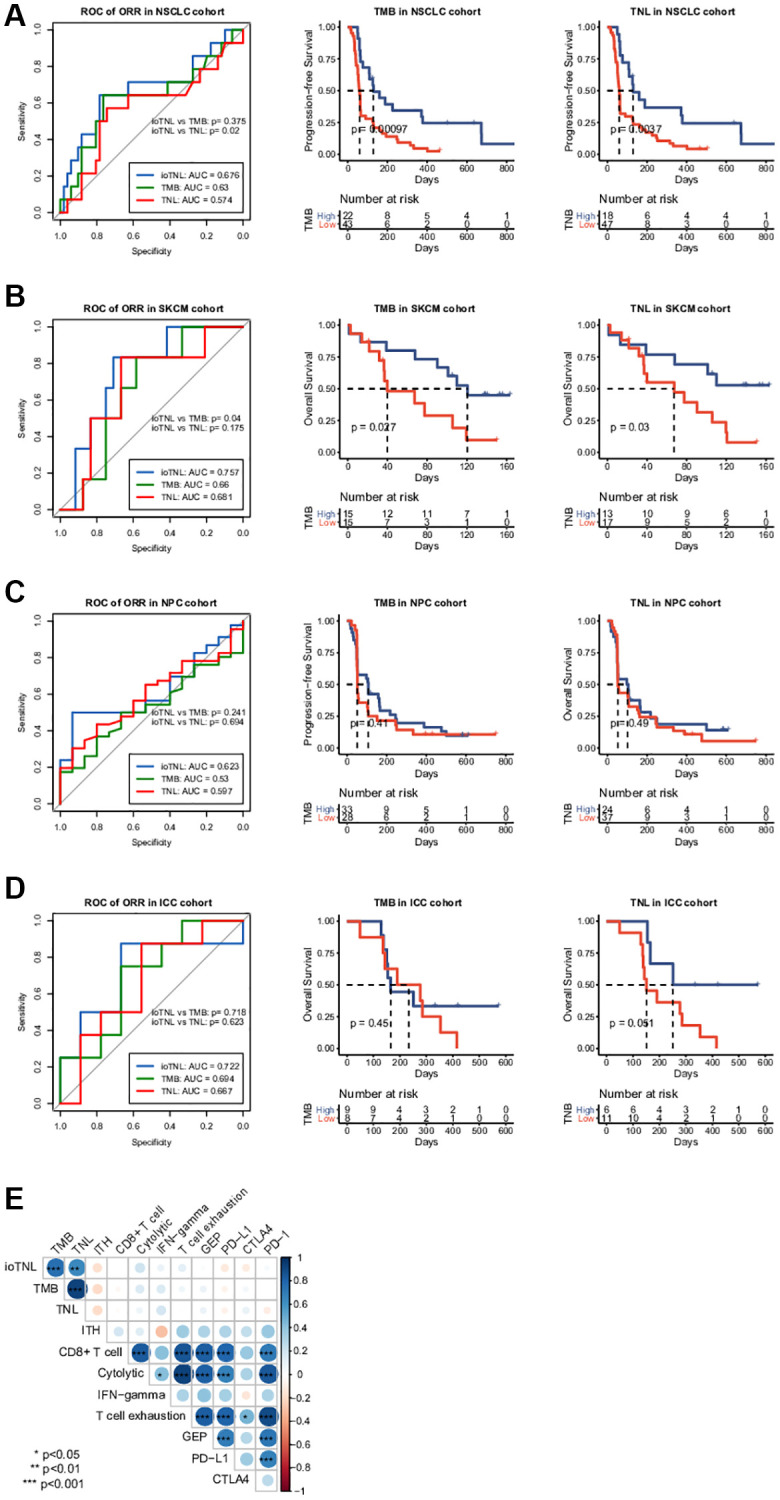
**Association between ioTNL score and other immunotherapy biomarkers.** Comparison of the response prediction among TMB, TNL and ioTNL in the NSCLC cohort (**A**, left), the SKCM cohort (**B**, left), the NPC cohort (**C**, left) and the ICC cohort (**D**, left). Kaplan-Meier analysis of patient survival of TMB and TNL in the NSCLC cohort (**A**, middle and right), the SKCM cohort (**B**, middle and right), the NPC cohort (**C**, middle and right) and the ICC cohort (**D**, middle and right). (**E**) Spearman correlation of ioTNL with genomic biomarkers and microenvironment-related biomarkers in SKCM cohort. Positive correlations are labeled in blue and negative correlation are labeled in red. Size of circle represented the value of correlation.

We analysed the association between TMB as well as TNL and clinical outcomes to compare the predictive performance of ioTNL with known genomic biomarkers of immunotherapy (Supplementary Figure 3). The TMB could significantly predict response only in the NSCLC cohort (*p* = 0.01, Supplementary Figure 3A, left). Similarly, TNL could also predict response only in the NSCLC cohort (*p* = 0.0475, Supplementary Figure 3A, right). In the other three cohorts, patients with a high TMB or a high TNL had a tendency of better response; however, the outcomes were not significant (Supplementary Figure 3B–3D). We used the ROC curve to measure the predictive efficiency in the four cohorts. We found that the AUC of the ioTNL was greater than that of the TMB and TNL in all four cohorts (Figure 4A–4D, left). Following this, the association between the TMB as well as TNL and survival were analyzed. TMB could predict survival in two cohorts, namely, NSCLC (*P* < 0.001, Figure 4A, middle) and SKCM (*p* = 0.027, Figure 4B, middle). Similarly, TNL could predict survival in the NSCLC (*p* = 0.0037, Figure 4A, right) and the SKCM cohorts (*p* = 0.03, Figure 4B, right). However, the ioTNL could predict survival in all four cohorts and had more significant *p* values (Figure 3A–3D). The association between biomarkers of the microenvironment and the ioTNL was compared in the SKCM cohort. Immune checkpoint genes (PD-1, PD-L1 and CTLA4), immune cell abundance (CD8+ T cell and CD4+ T cell) as well as immune signatures (cytolytic, IFN-gamma and T cell exhaustion signatures) were identified from RNA transcriptome data. None of these microenvironment biomarkers correlated with the ioTNL (Spearman correlation <0.2, Figure 4E). Taken together, our data demonstrated that the ioTNL is a better genomic biomarker than the TMB and TNL in predicting the efficacy of immunotherapy. In addition, the ioTNL is independent of the microenvironment biomarkers.

### Panel-based immune editing score correlated with the TMB and ORR of immunotherapy in different cancers

**Figure 5:**
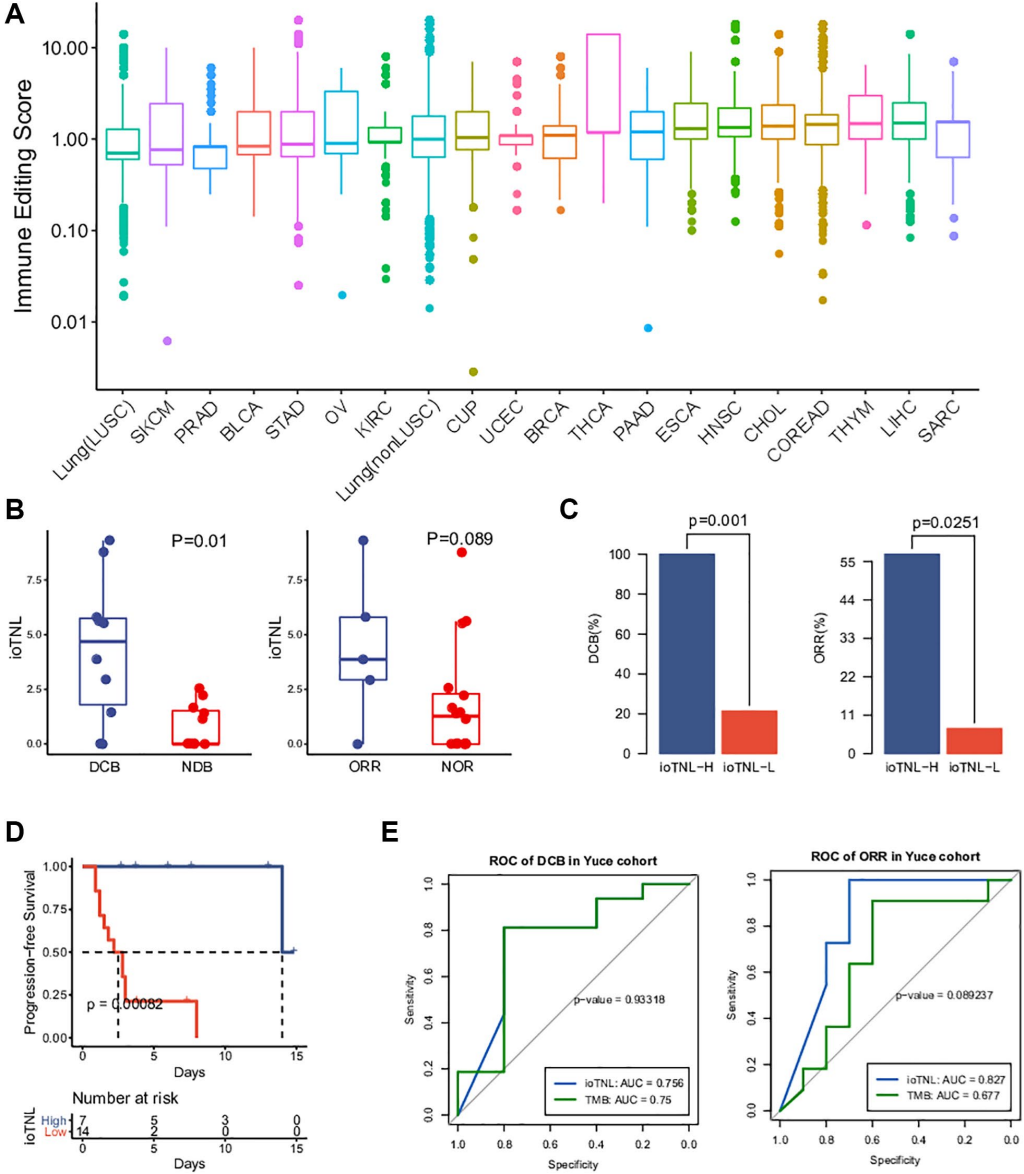
**Landscape of immune editing score and application of ioTNL on panel-based Yuce cohort.** (**A**) Landscape of immune editing score in 20 cancer types. The immune editing scores were scale in log10 on y-axis. 20 cancer types were label on x-axis and arranged in ascending order by their median immune editing score. (**B**) Boxplots of the distribution of ioTNL scores between patients with DCB and NDB (left), also with ORR and NOR (right). (**C**) Barplots of DCB rate (left) and ORR rate (right) between ioTNL-H group and ioTNL-L group. (**D**) Kaplan-Meier analysis of patient progression-free survival between ioTNL-H group and ioTNL-L group. (**E**) Comparison of sensitivity and specificity between ioTNL and TMB in predicting patient DCB (left) and ORR (right).

Compared with WES, target sequencing on an appropriate region size of the panel would be a cost-effective alternative for clinical detection. To facilitate the application of immunoediting status to clinical detection and the assessment of the ioTNL, we designed YuceOne, a 1.4M panel assembled by screening genomic regions and HLA genes that are susceptible to neoantigens. We collected the data of 2,469 patients across 20 cancer types and applied them on YuceOne to detect mutations and predict neoantigens (Supplementary Table 6). The same approach was followed to calculate the immune editing scores in each clone cluster for each patient. We demonstrated the landscape of immune editing scores across 20 cancer types and found that the majority of the clones had scores ranging from 0 to 10, with a median value of approximately 1 (Figure 5A). Carcinomas such as LUSC, SKCM, PRAD, BLCA and STAD had lower median immune editing scores than 1, implying stronger immune clearance in the clones. On the other hand, carcinomas such as SARC, LIHC and THYM had higher median immune editing scores than 1, implying more severe immune escape in the clones (Figure 5A).

A previous study reported a significant correlation between the median TMB and the ORR following anti–PD-(L)1 therapy [1]. To explore whether the immune editing score has a similar correlation with the TMB and ORR, we collected the median TMB and response data of ICI monotherapy for each cancer type. We plotted the median TMB and the ORR for anti–PD-(L)1 therapy against the corresponding median immune editing score across multiple cancer types (Supplementary Table 7). There was a significant negative correlation between the immune editing score and the TMB (R = −0.54, *p* = 0.014, Supplementary Figure 4A). Carcinomas with a high TMB such as SKCM, LUSC and BLCA had lower median immune editing scores, indicating stronger immune clearance in the tumor clones. We also observed a similar tendency of negative correlation between the immune editing score and clinical response; however, the outcomes were not significant (R = −0.45, *p* = 0.12, Supplementary Figure 4B). The lower the median immune editing score in one cancer type, the higher the ORR for its anti–PD-(L)1 therapy.

### ioTNL identified by the target panel predicted the clinical outcome of immunotherapy

The datasets used for evaluating and verifying the ioTNL were derived from WES. To expand the application of the ioTNL model and explore whether it can predict the response to immunotherapy on a panel level, we collected the data of 21 patients with NSCLC with complete clinical outcomes who underwent anti-PD-1/PD-L1 therapy and identified the ioTNL using YuceOne. Consistent with the previously reported findings, the ioTNL scores could successfully predict the response to immunotherapy (Supplementary Table 8). Patients with a DCB and ORR showed a higher ioTNL (Figure 5B). Patients in the ioTNL-high group, had a significant DCB (100% vs. 21%, *p* = 0.001, Figure 5C) and ORR (57% vs. 7%, *p* = 0.025, Figure 5D) compared with those in the ioTNL-low group. Meanwhile, PFS analysis revealed that patients with higher ioTNL had a longer survival time (*P* < 0.001, Figure 5D). For comparison, the TMB and TNL were also evaluated in our cohort (Supplementary Table 2), and the ROC curves showed that the ioTNL performed better in predicting the response to immunotherapy (Figure 5E).

## DISCUSSION

We developed an algorithm named ioTNL optimised from the tumor neoantigen load based on the immune editing stages. The ioTNL was related to the response and survival in NSCLC, SKCM, melanoma, ICC and NPC. Our data demonstrated that ioTNL could be a prediction model for checkpoint inhibitors, which is more robust than the traditional neoantigen count model and the TMB.

Compared with an assessment of the TMB, relatively few studies have revealed the correlation between the TNL and clinical outcomes. In these studies, the TNL was usually worse than the TMB in predicting clinical efficacy, likely due to the high false-positive rate of neoantigen prediction. Another reason might be that neoantigens are heterogeneous in tumors. The information on neoantigen clones would be ignored by directly counting the number of neoantigens. Some algorithms, such as DAI, focus on identifying neoantigen profiling with high immunogenicity and demonstrate the difference in the predicted affinity for any given wild-type/mutant peptide pair [14]. Similarly, the fitness model selects the neoantigen with the highest immunogenicity from missense derived to determine the sufficient size of a cancer cell population. Some studies have reported that clonal but not subclonal neoantigens are associated with patient survival, such as the clonal neoantigen burden, DAI and CSiN [15]. The studies optimized the neoantigen quantification method based on the quality and clonality; however, they did not consider immune editing during the evolution of the tumor and host immune microenvironment.

Here, we proposed a direction for improvement based on the neoantigen immunoediting stages. If the tumor clone has been immune edited and lost the ability of immune elimination, the possibility of recognising and eliminating these immune tolerant neoantigens will be reduced. Neoantigens in immune edited clones do not contribute to the immunogenicity of tumors. An extensive analysis of a patient with metastatic colorectal cancer during the 11 years of spatiotemporal follow-up showed that the persistent clone had a higher immunoediting score than the eliminated clone [16]. Depletion of neoantigens has been observed in microsatellite instability (MSI) colorectal cancer, known to be sensitive to ICIs [17]. These studies support our hypothesis that tumor clones designated as immunoedited had immune privilege despite the presence of tumor-infiltrating immune cells. Therefore, these clones were resistant to checkpoint inhibitors, and only clones with elimination capability were sensitive to the drugs. Tumor immunoediting influences the intratumoral heterogeneity and shapes clonal evolution.

Additionally, we optimised the algorithm of immunoediting score calculation. A former study reported that the ratio of the neoantigen to non-silent mutation could be calculated to evaluate the immunoediting status [18]. It represents the ratio of the expected to observed immunogenic mutations per non-silent mutation, which is highly dependent on the background mutation reference sets for the expected neoantigen rate. Therefore, we assessed the prediction efficiency with and without reference sets. We found that the model without a reference set demonstrated best practice performance (Supplementary Figure 5). It has been reported that the neoantigens caused by InDels have higher immunogenicity than those from missense mutations [19]. We also considered InDel-derived neoantigens in the ioTNL algorithm, except for missense-derived neoantigens. Another optimisation is that the ioTNL enriches truncal neoantigens by calculating the total cancer cell fraction as the neoantigen concentration instead of the neoantigen load of tumor clones.

Assessment of the ioTNL could be more suitable for clinical application. In one way, the ioTNL is an RNA-independent method. The expression of mutated genes is essential for neoantigen prediction. Some algorithms, such as the fitness model and CSiN, need transcriptome data to determine whether the mutation is expressed. We used TCGA data instead of transcriptome sequencing to predict the expression level of genes. We assessed the prediction performance of the ioTNL model by comparing models with or without RNA sequencing data to evaluate the expression level of the neoantigens in the SKCM cohort. We found that the performance of the algorithm without RNA sequencing data was not inferior to that with RNA sequencing data (Supplementary Figure 6). Obtaining RNA tissue specimens of high quality is a significant challenge in clinical practice, especially for formalin-fixed paraffin-embedded (FFPE) samples. Such optimisation not only improves the convenience but also reduces the sequencing cost to apply the ioTNL. Furthermore, the ioTNL was validated through the multiple genes targeted panel. This panel was designed to capture HLA genes and regions that enrich neoantigens rather than hotspot mutations. The targeted panel was more suitable for clinical application than WES was due to the low specimen input and cost.

There are some limitations to our study. Although we validated the ioTNL in four different cohorts, including the main cancer types for immune checkpoint therapy (NSCLC and SKCM), the scale of the validation cohort remains limited. More cancer types should be covered in the future, not only including cancers with high load neoantigens (i.e., NSCLC and SKCM) but also those with median load neoantigens (i.e., ICC and NPC). Recent studies revealed that genomic biomarkers such as the TMB failed to predict the clinical outcome of combination immunotherapy. The ioTNL had a better predictive ability than the TMB and TNL in our data. However, the ioTNL needs to be verified as a genomic biomarker for predictive performance in combination immunotherapy, combined with microenvironmental biomarkers, such as PD-L1, IFN-gamma signature and T cell infiltration. Finally, the accurate identification of neoantigens is the supreme challenge for the ioTNL algorithm, which is also a common challenge in the research on neoantigens. We believe the addition of mass spectrometry data would improve the accuracy of identifying the neoantigens by applying artificial intelligence technology.

In conclusion, we developed a neoantigen algorithm based on immune editing to evaluate the response and prognosis of ICIs. We validated the prediction performance of the ioTNL in four cancers, including NSCLC, SKCM, ICC and NPC. The ioTNL predicted the response and prognosis better than the TMB and TNL. The multiple genes targeted panel was developed for ioTNL testing and can be considered to personalise immunotherapy. The immunoediting stages integrated into the ioTNL should also be considered in neoantigen selection for adoptive cell transfer and vaccine development.

## METHODS

### Mutation calling

After removing the reads containing sequencing adapters and low-quality reads with more than five ambiguous bases, high-quality reads were aligned to the NCBI human reference genome (hg19) using BWA (v0.5.9) [20] with default parameters. Picard (v1.54) [21] (http://picard.sourceforge.net/) was used to mark duplicates, followed by the use of the Genome Analysis Toolkit [22] (v1.0.6076, GATK IndelRealigner) to improve alignment accuracy.

Somatic SNVs were detected by VarScan2.2.5 [23] based on the BWA align algorithm and high-confidence somatic SNVs were called if the following criteria were met: (I) both the tumor and normal samples were covered sufficiently (≥ 10×) at the genomic position; (II) the variants were supported by at least 5% of the total reads in the tumor in contrast to less than 2% in the normal; (III) the variants were supported by at least three reads in the tumor; (IV) the distance between adjacent somatic SNVs were over 10 bp; (V) the mapping qualities of reads supporting the mutant allele in the tumor were significantly higher than 30 (Wilcoxon rank sum test, *P* < 0.2); (VI) the base qualities of reads supporting the mutant allele in the tumor were significantly higher than 20 (Wilcoxon rank sum test, *P* < 0.05); (VII) the mutations were not enriched within the 5 bp 5’ or 3’ of the read end (Wilcoxon rank sum test, *P* < 0.1); (VIII) the mutant allele frequency changes between the tumor and blood were statistically significant (Fisher’s exact test, *P* < 0.05).

High-confidence somatic InDels were called based on the following steps: (I) candidate somatic InDels were predicted with the GATK somatic InDel detector with default parameters; (II) for each predicted somatic InDel, local realignment was performed with combined normal and tumor bam files; (III) frequent of variant reads less than 10% were filtered. (IV) high-confidence somatic InDels were defined after filtering germline events.

Finally, all mutations were re-annotated using in-house annotation software based on SnpEff [24].

### Copy number analysis and tumor purity assessment

Copy number variants (CNV) were called by exome-wide profile comparisons between tumors and matched peripheral blood using CNVkit (v0.8.1). Allele-specific copy number and tumor purity of the tumor genome were assessed using ascatNgs (v3.1.0).

### HLA class I neoantigen predictions, prioritization, and selection

HLA genotype is identified with combination use of Polysover [25] and BWA-HLA. 9-mer polypeptides centered on mutated residues were scanned to identify candidate peptides binding to HLA class I, i.e., peptide sequences surrounding mutated amino acids resulting from missense mutations, frame-shift or non-frame-shift indels. The affinity of 8–11 mer peptides binding to HLA class I was predicted using the NetMHCPan4.0 [26] binding algorithm.

### Tumor clone determination

PyClone (v0.13.0) was used to infer the cancer cell fraction (CCF) of mutations in tumors. Major copy number and minor copy number of each mutation were acquired from the result of ascatNgs software. Moreover, set prior to major_copy_number. For some samples whose copy number of each SNV and tumor purity information were not accessible, those parameters were set to the default value and set prior to total_copy_number. PyClone was deployed with 10,000 iterations and a burn-in of 1000 for all samples.

### Calculation of immune editing score and ioTNL score

ioTNL was calculated by counting neoantigen concentration selected from tumor clones which could induce immune elimination. We calculated the ratio of neoantigen to non-silent mutation as immune editing score to quantify the immune editing stages for tumor clones.


$$Immune\;editing\;score=\frac{n_{neoantigen}}{n_{nonsilent\;mutation}}$$

Tumor clonal architecture was calculated by PyClone. Cancer cell fraction of each neoantigen in the immune elimination clone were sum up as final ioTNL.


$$ioTNL=\sum_{i=1}^{n}load_{i}\times CCF_{i}$$

Where *n* represents the total number of immune elimination clone, load_i_ is the number of neoantigens in the immune elimination clone i and CCF_i_ is the cancer cell fraction in the immune elimination clone i. We found the best cutoff of immune editing score from 0.5 to 1.5 in increment of 0.1 when the best prediction performance of ioTNL was found. The best prediction performance of ioTNL was found as maximum area of ROC curve. The cutoff of ioTNL was identified by Youden index (the maximum sensitivity and the best specificity under the AUC curve).

### Statistical analysis

Categorical variables were evaluated with Fisher’s exact tests. Unpaired Mann–Whitney *U* test was used to compare differences for continuous variables between groups. Correlation analysis was assessed by Pearson coefficient. ROC analysis was done using the ROCR package in R. Significance of overall survival (OS) and progression-free survival (PFS) was determined via Kaplan-Meier analysis with log-rank analysis. The hazard ratio was calculated by the coxph function of the survival package in R. All statistical analysis was performed in the R statistical environment version 3.6.1. All tests were two-tailed and *p*-value < 0.05 was considered significant.

### Ethics approval and consent to participate

A 68-year-old squamous cell carcinoma of the lung cancer patient01 was enrolled in Shanghai Tenth People’s Hospital on 2018. The study was approved by Shanghai Tenth People’s Hospital ethics committee. The patient provided written informed consent.

### Availability of data and material

All of the published data used in the article can be found on the NCBI website and supplementary material, all the material used in the experiment can be bought. Codes and YuceOne (panel-based) data are available upon reasonable request.

## Supplementary Materials

Supplementary Figures

Supplementary Tables 1, 3, 5, 7 and 8

Supplementary Tables 2 and 4

Supplementary Table 6
