# Supplementary material for: Construction and validation of an immunoediting-based optimized neoantigen load (ioTNL) model to predict the response and prognosis of immune checkpoint therapy in various cancers
Source: Aging (Albany NY). 2022 May 25;14(10):4586–605. doi: 10.18632/aging.204101 (PMC9186755; doi:10.18632/aging.204101)
Supplement: Supplementary Figures [file aging-14-204101-s001.pdf]

SUPPLEMENTARY FIGURES

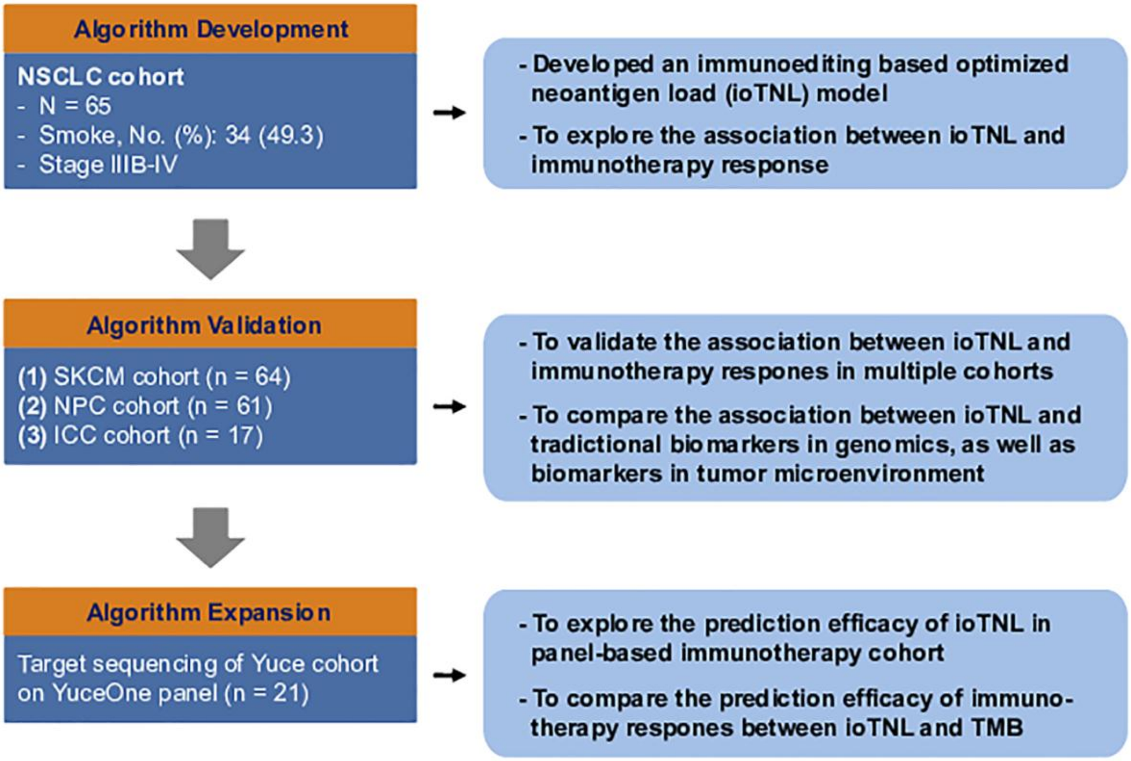

Supplementary Figure 1. The study diagram of the ioTNL model.

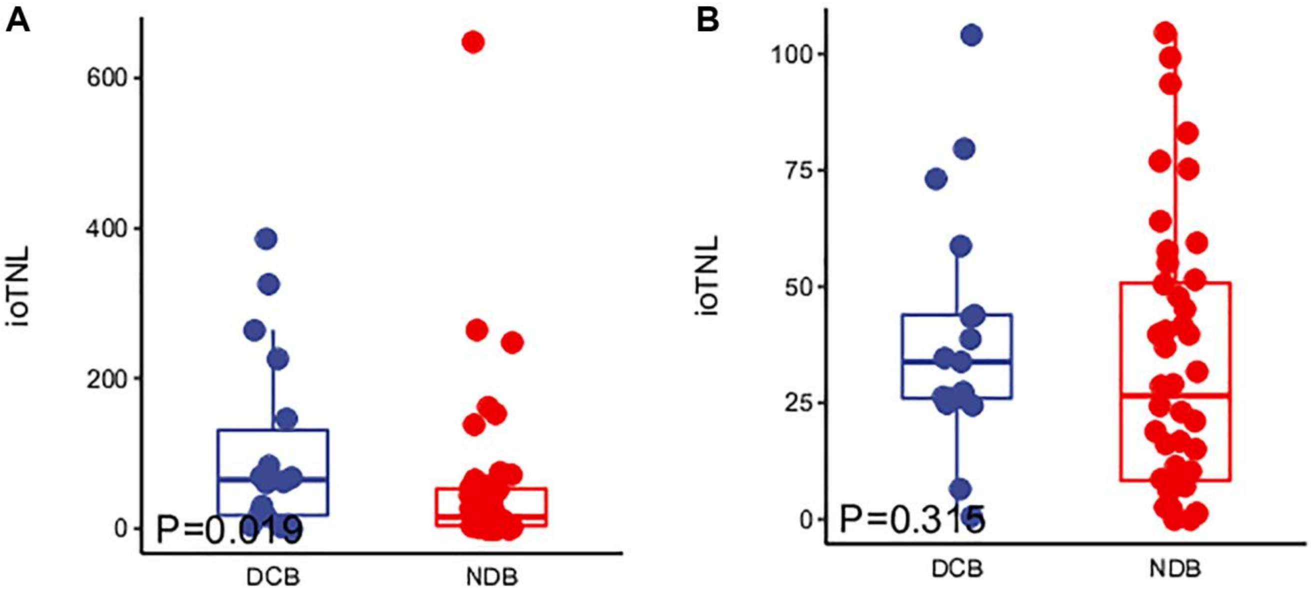

Supplementary Figure 2. Boxplots of the distribution of ioTNL scores between patients with DCB and NDB in the NSCLC cohort (**A**) and the NPC cohort (**B**).

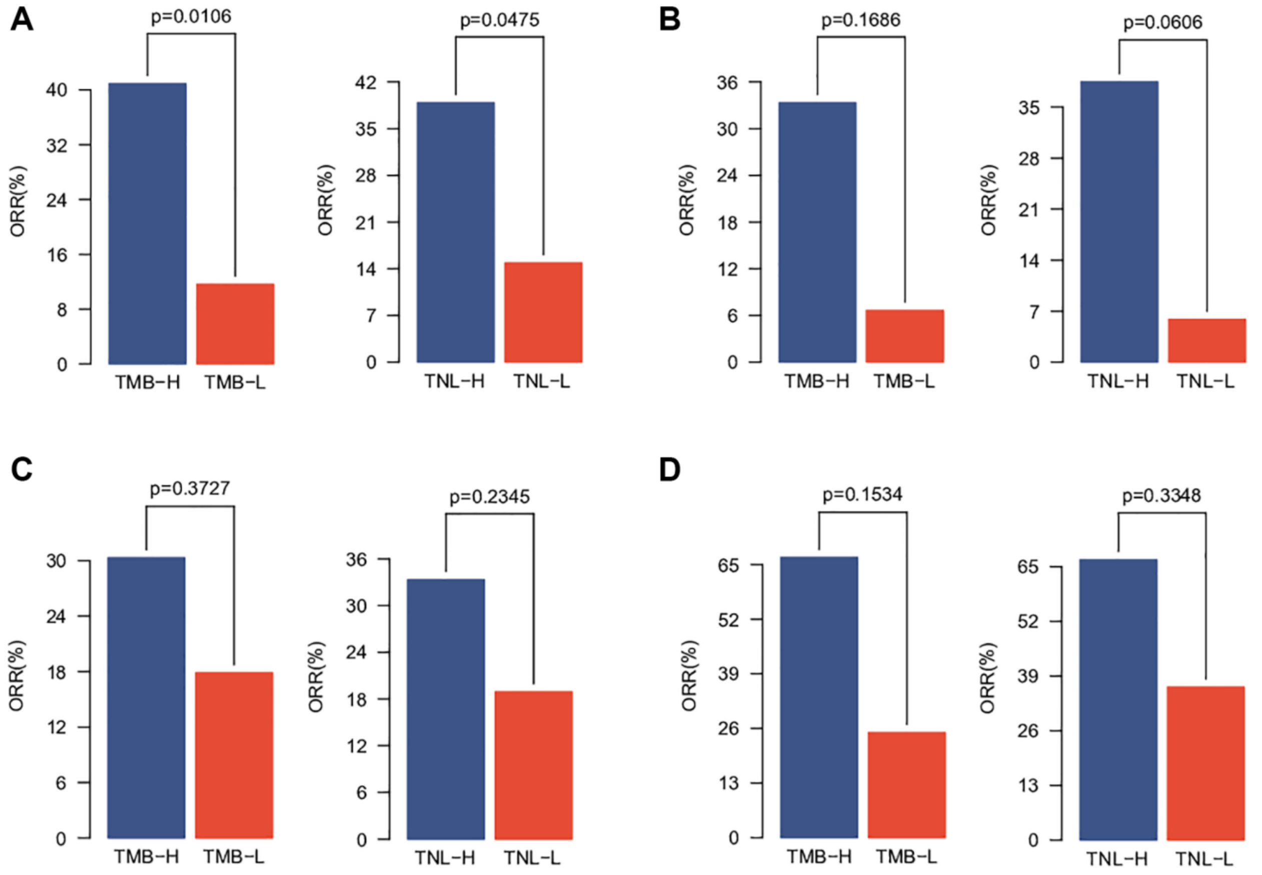

Supplementary Figure 3. Barplots of ORR rate of different TMB levels and TNL levels in the NSCLC cohort (**A**), the SKCM cohort (**B**), the NPC cohort (**C**) and the ICC cohort (**D**).

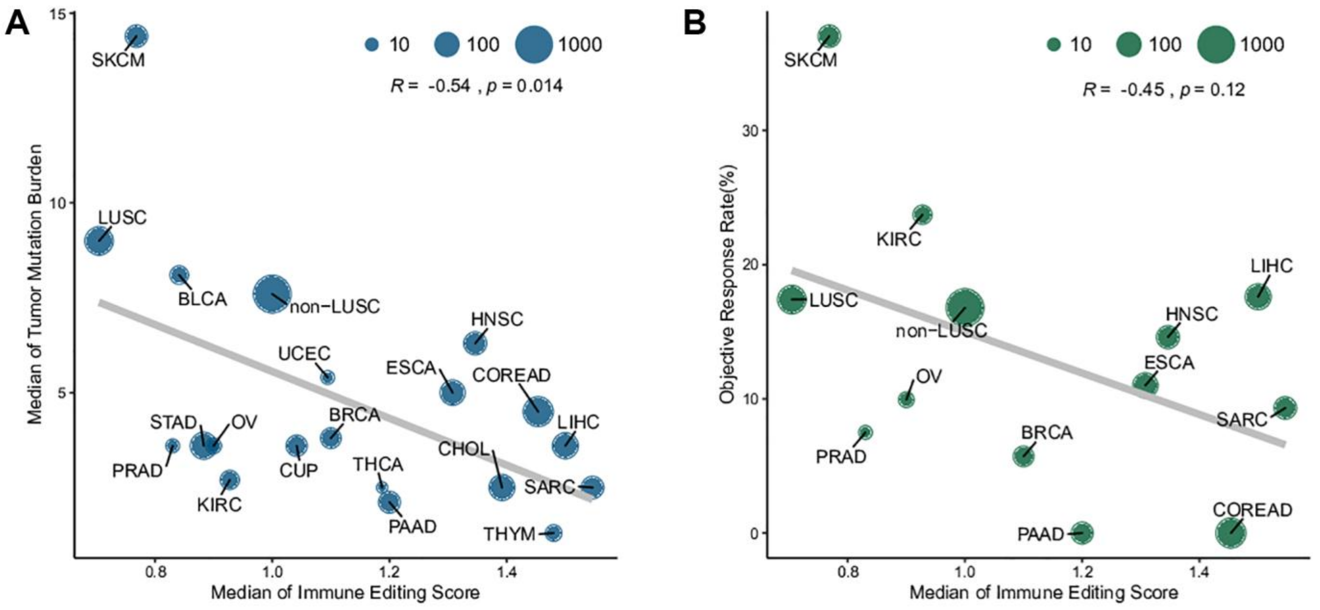

Supplementary Figure 4. Immune editing score was associated with TMB and objective response rate of immunotherapy. (**A**) Correlation between median immune editing score and median TMB in 20 cancer types. (**B**) Correlation between median immune editing score and objective response rate of immunotherapy in multiple cancer types. Size of circle represents the number of patients that involved in the evaluation of immune editing score.

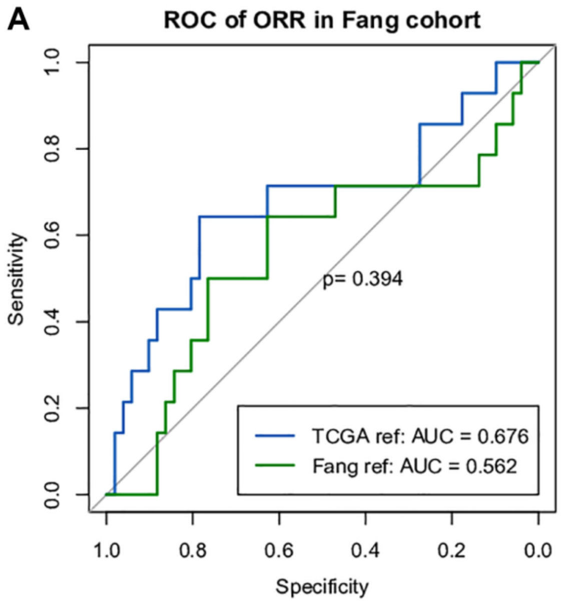

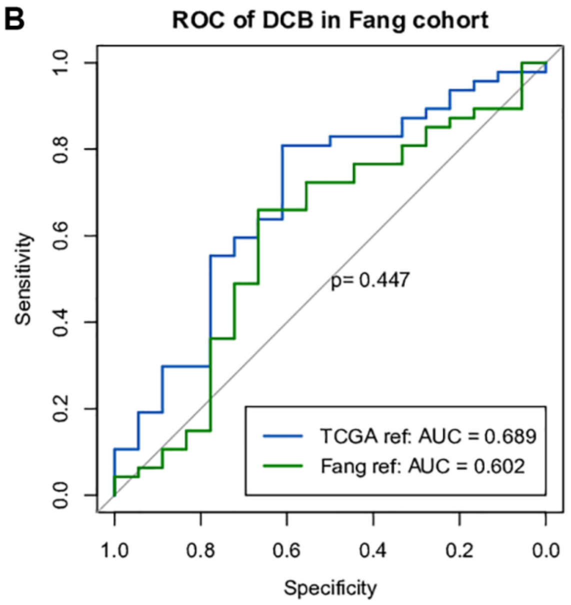

Supplementary Figure 5. Prediction efficiency with different reference set of ORR (**A**) and DCB (**B**) in the NSCLC cohort.

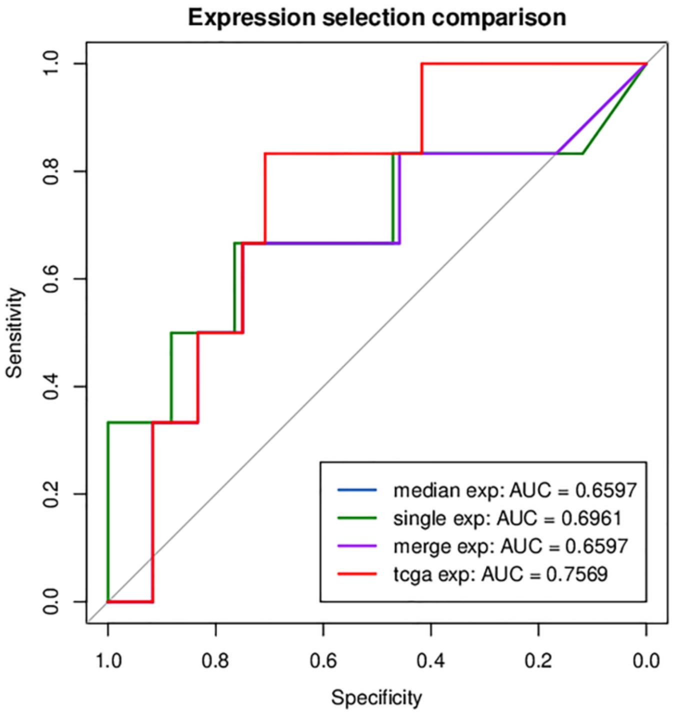

Supplementary Figure 6. Prediction efficiency with different gene expression dataset.
