# Supplementary material for: Construction and validation of an immunoediting-based optimized neoantigen load (ioTNL) model to predict the response and prognosis of immune checkpoint therapy in various cancers
Source: Aging (Albany NY). 2022 May 25;14(10):4586–605. doi: 10.18632/aging.204101 (PMC9186755; doi:10.18632/aging.204101)
Supplement: Supplementary Tables 1, 3, 5, 7 and 8 [file aging-14-204101-s002.pdf]

Supplementary Tables

Supplementary Table 1. Detailed information of the introduced immunotherapy cohorts.

Cohort	Cohort size	Histology	Treatment	Sequence type	References
NSCLC cohort	65	Non-small-cell lung cancer	Anti-PD-(L)1	WES	Fang et al., 2019
SKCM cohort	64	Skin cutaneous melanoma	Anti-PD-1	WES	Riaz et al., 2017
NPC cohort	61	Nasopharyngeal carcinoma	Anti-PD-(L)1	WES	Fang et al., 2018
ICC cohort	17	Intrahepatic cholangiocarcinoma	Anti-PD-1 + chemotherapy	WES	Feng et al., 2020
Yuce cohort	21	Non-small-cell lung cancer	Anti-PD-1	Panel	–
Total	228				

Supplementary Table 3. Summary of ioTNL and clinical characteristics in SKCM cohort.

Patient	Sample	TMB	TNL	ioTNL	Treated	Response	ORR	OS	Status
PT100	PT100-BE	0.13	0.1	0	NIV3-PROG	PD	NOR	120	1
PT101	PT101-BE	0.1	0.07	0	NIV3-PROG	PR	ORR	119.1	0
PT103	PT103-BE	0.26	0.19	0	NIV3-PROG	PD	NOR	69.1	0
PT104	PT104-BE	0.16	0.1	0	NIV3-PROG	PR	ORR	116.9	0
PT106	PT106-BE	18.59	7.89	340.327	NIV3-PROG	PD	NOR	13	1
PT108	PT108-BE	0.62	0.16	3.098	NIV3-PROG	SD	NOR	130.4	0
PT10	PT10-BE	2.48	0.89	28.9125	NIV3-NAIVE	SD	NOR	36.6	1
PT11	PT11-BE	2.3	1.26	47.0899	NIV3-NAIVE	PD	NOR	119.6	1
PT13	PT13-BE	4.48	2.08	82.1663	NIV3-NAIVE	PD	NOR	40	1
PT17	PT17-BE	0.16	0.16	0	NIV3-PROG	PD	NOR	8.1	1
PT18	PT18-BE	4.31	2.5	92.5359	NIV3-NAIVE	PR	ORR	153.3	0
PT24	PT24-BE	0.1	0.03	0	NIV3-NAIVE	PD	NOR	21.3	0
PT25	PT25-BE	2.27	1.36	0	NIV3-NAIVE	PD	NOR	14.4	1
PT27	PT27-BE	4.69	2.88	0	NIV3-NAIVE	PD	NOR	67.9	1
PT28	PT28-BE	1.2	0.68	7.0901	NIV3-NAIVE	PD	NOR	105.7	1
PT29	PT29-BE	11.54	7.17	0.5179	NIV3-NAIVE	PD	NOR	39	1
PT30	PT30-BE	1.82	0.42	18.1944	NIV3-NAIVE	CR	ORR	150.4	0
PT31	PT31-BE	10.86	5.48	166.906	NIV3-NAIVE	PD	NOR	137.3	0
PT32	PT32-BE	3.2	1.45	56.7156	NIV3-PROG	PD	NOR	16.3	0
PT34	PT34-BE	3.77	2.63	107.228	NIV3-PROG	PR	ORR	119.1	0
PT36	PT36-BE	0.19	0.1	0	NIV3-PROG	SD	NOR	154.4	0
PT37	PT37-BE	0.57	0.27	3.73814	NIV3-PROG	SD	NOR	92.3	1
PT38	PT38-BE	9.4	4.56	94.1405	NIV3-PROG	SD	NOR	23.9	1
PT3	PT3-BE	4.93	2.24	98.4519	NIV3-NAIVE	PR	ORR	163.4	0
PT44	PT44-BE	10.39	4.88	274.331	NIV3-NAIVE	PR	ORR	156.1	0
PT46	PT46-BE	4.02	2.43	0	NIV3-PROG	PD	NOR	32.4	1
PT47	PT47-BE	24.8	12.64	535.166	NIV3-PROG	PD	NOR	102.6	1

PT48	PT48-BE	1.83	0.93	21.0797	NIV3-PROG	CR	ORR	149.4	0
PT4	PT4-BE	6.9	1.78	97.4983	NIV3-NAIVE	SD	NOR	90.4	1
PT51	PT51-BE	5.91	2.5	93.7713	NIV3-PROG	PD	NOR	8.1	0
PT52	PT52-BE	7.68	4	130.458	NIV3-PROG	PD	NOR	68	1
PT53	PT53-BE	2.08	0.52	14.6259	NIV3-PROG	PR	ORR	61.9	1
PT58	PT58-BE	22.65	10.97	524.13	NIV3-PROG	SD	NOR	71.3	1
PT59	PT59-BE	9.58	3.77	127.405	NIV3-NAIVE	SD	NOR	101.1	1
PT5	PT5-BE	0.85	0.36	6.06482	NIV3-NAIVE	PD	NOR	31.7	1
PT60	PT60-BE	17.36	8.07	451.967	NIV3-NAIVE	PD	NOR	105.4	0
PT66	PT66-BE	3.94	1.7	72.4464	NIV3-NAIVE	PD	NOR	77.4	1
PT67	PT67-BE	0.45	0.26	0	NIV3-PROG	SD	NOR	147.4	0
PT68	PT68-BE	19.34	9.46	382.127	NIV3-PROG	PR	ORR	120.3	0
PT70	PT70-BE	11.3	7.72	417.55	NIV3-PROG	SD	NOR	44.4	1
PT71	PT71-BE	0.35	0.13	0	NIV3-NAIVE	SD	NOR	35.1	0
PT72	PT72-BE	12.11	5.76	262.428	NIV3-NAIVE	PR	ORR	110	1
PT73	PT73-BE	0.45	0.1	1.02686	NIV3-NAIVE	SD	NOR	40.1	0
PT74	PT74-BE	1.48	0.75	32.3266	NIV3-NAIVE	NE	NOR	2.9	1
PT76	PT76-BE	6.55	3.21	124.572	NIV3-NAIVE	NE	NOR	1.4	1
PT77	PT77-BE	1.11	0.49	6.35038	NIV3-NAIVE	SD	NOR	67.3	1
PT79	PT79-BE	15.62	8.46	409.153	NIV3-PROG	SD	NOR	31.1	1
PT82	PT82-BE	1.19	0.42	17.9164	NIV3-PROG	SD	NOR	61.3	1
PT83	PT83-BE	8.3	2.51	79.4521	NIV3-PROG	SD	NOR	66	0
PT84	PT84-BE	0.57	0.37	2.72251	NIV3-NAIVE	PD	NOR	21.6	1
PT85	PT85-BE	3.45	1.74	98.2943	NIV3-PROG	PD	NOR	129.1	0
PT86	PT86-BE	14.85	8.33	353.337	NIV3-PROG	PD	NOR	38.9	1
PT87	PT87-BE	17.14	8.34	383.341	NIV3-NAIVE	SD	NOR	139.1	0
PT89	PT89-BE	4.81	1.77	83.7375	NIV3-NAIVE	SD	NOR	120.6	1
PT8	PT8-BE	2.38	1.02	54.6892	NIV3-NAIVE	PD	NOR	37	1
PT90	PT90-BE	8.72	4.33	132.597	NIV3-PROG	PD	NOR	24.9	1
PT92	PT92-BE	16.27	6.78	225.266	NIV3-PROG	SD	NOR	47.6	1
PT93	PT93-BE	0.81	0.46	11.4005	NIV3-PROG	PD	NOR	121.3	0
PT94	PT94-BE	9.19	4.93	141.264	NIV3-NAIVE	CR	ORR	140.1	0
PT98	PT98-BE	0.59	0.33	10.0383	NIV3-PROG	SD	NOR	106.7	1
PT9	PT9-BE	11.13	3.88	166.344	NIV3-NAIVE	PD	NOR	13.1	1

Supplementary Table 5. Summary of ioTNL and clinical characteristics in ICC cohort.

Sample	TMB	TNL	ioTNL	Response	ORR	OS	OS.Status	PFS	PFS.Status
ICC001	2.85	1.39	69.4077	PR	ORR	334	0	NA	0
ICC002	2.96	1.12	37.032	PR	ORR	151	1	114	1
ICC003	1.33	0.65	14.3812	SD	NOR	415	1	236	1
ICC004	4.65	2.03	80.6436	CR	ORR	572	0	324	1
ICC005	4.5	1.5	50.0041	SD	NOR	154	1	86	1
ICC006	2.56	1.37	14.8543	SD	NOR	143	1	129	1
ICC007	3.24	1.36	48.5457	PD	NOR	138	1	45	1
ICC008	0.94	0.25	1.94014	SD	NOR	277	1	172	1
ICC009	1.91	0.5	4.8922	SD	NOR	353	1	230	1
ICC010	1.46	0.94	9.91282	PD	NOR	136	1	66	1
ICC011	3.37	1.63	57.3618	PR	ORR	165	1	82	1
ICC012	1.63	0.53	17.841	PR	ORR	285	1	NA	0
ICC013	6.28	2.24	82.7309	PR	ORR	420	0	NA	0
ICC014	4.46	2.26	74.1739	SD	NOR	251	1	144	1
ICC015	1.39	0.97	0	PR	ORR	190	1	92	1
ICC016	1.3	0.68	0.29386	SD	NOR	49	1	47	1
ICC017	2.94	1.23	36.0288	PR	ORR	129	1	117	1

Supplementary Table 7. Median immune editing score and corresponded median TMB and ORR of immunotherapy in pancancer.

OncoTree	Median Immune Editing Score	Median TMB	ORR	# of Patients
BLCA	0.841269841	8.1	NA	28
BRCA	1.1	3.8	5.7149	41
CHOL	1.391891892	2.5	NA	114
COREAD	1.453608247	4.5	0	254
CUP	1.041666667	3.6	NA	46
ESCA	1.307692308	5	11	99
HNSC	1.346153846	6.3	14.5987	65
KIRC	0.927536232	2.7	23.7273	31
LIHC	1.5	3.6	17.6074	115
LUSC	0.704545455	9	17.402	186
non-LUSC	1	7.6	16.7981	1116
OV	0.9	3.6	9.93794	16
PAAD	1.2	2.112786	0.0006	52
PRAD	0.830188679	3.6	7.49976	11
SARC	1.546391753	2.5	9.32155	60
SKCM	0.768421053	14.4	37.039	58
STAD	0.883116883	3.6	NA	140
THCA	1.1875	2.5	NA	7
THYM	1.48	1.3	NA	19
UCEC	1.094594595	5.4	NA	11

Supplementary Table 8. Summary of ioTNL and clinical characteristics in Yuce cohort.

Sample	TMB	ioTNL	Benefit	DCB	ORR	PFS	Status
Yuce001	3.36	0	PD	NDB	DOR	0.9	1
Yuce002	8.73	0	PD	NDB	DOR	2.2	1
Yuce003	16.13	0	PD	NDB	DOR	1.2	1
Yuce004	12.76	5.60849	SD	DCB	DOR	7.6	0
Yuce005	5.38	0	PD	NDB	DOR	2.8	1
Yuce006	18.13	9.29958	PR	DCB	ORR	3.7	0
Yuce007	8.06	1.15252	PD	NDB	DOR	1.2	1
Yuce008	1.34	0	SD	DCB	DOR	7.3	0
Yuce009	2.02	1.39332	PD	NDB	DOR	0.9	1
Yuce010	6.72	8.75306	SD	DCB	DOR	14	1
Yuce011	15.4	3.86614	PR	DCB	ORR	2.7	0
Yuce012	12.07	5.79855	CR	DCB	ORR	6	0
Yuce013	11.27	2.93277	CR	DCB	ORR	14.8	0
Yuce014	4.65	1.43219	SD	DCB	DOR	8	1
Yuce015	9.96	0	PD	NDB	DOR	3	1
Yuce016	6.64	1.64018	PD	NDB	DOR	2.8	1
Yuce017	12.61	5.50955	SD	DCB	DOR	13	0
Yuce018	1.34	0	PD	NDB	DOR	1.5	1
Yuce019	3.35	0	PR	DCB	ORR	3.8	0
Yuce020	4.02	2.21275	PD	NDB	DOR	3	1
Yuce021	5.36	2.54265	PD	NDB	DOR	1.8	1
