# Supplementary material for: Construction and validation of an immunoediting-based optimized neoantigen load (ioTNL) model to predict the response and prognosis of immune checkpoint therapy in various cancers
Source: Aging (Albany NY). 2022 May 25;14(10):4586–605. doi: 10.18632/aging.204101 (PMC9186755; doi:10.18632/aging.204101)
Supplement: Supplementary Tables 2 and 4 [file aging-14-204101-s003.docx]

| **Supplementary Table 2. Summary of ioTNL and clinical characteristics in NSCLC cohort.** | | | | | | | | | | | | | | | |
| --- | --- | --- | --- | --- | --- | --- | --- | --- | --- | --- | --- | --- | --- | --- | --- |
| **Patient ID** | **TMB** | **TNL** | **ioTNL** | **ITH** | **Smoke** | **Benefit** | **DCB** | **ORR** | **stat** | **PFS** | **histology** | **Stage** | **FarMetastasis** | **TreatLine** | **Drug** |
| F17120989176-KY438-VS-B17120989347-KY438 | 2.65 | 1.16 | 34.8697 | 0.93 | NEVER | PD | NDB | NOR | 1 | 43 | LUAD | NA | 3 | 2 | Nivolumab |
| F17120989180-KY438-VS-B17122392273-KY438 | 3.46 | 1.34 | 27.1891 | 0.97 | NEVER | PD | NDB | NOR | 1 | 35 | LUAD | NA | 2 | 2 | Nivolumab |
| F17120989185-KY438-VS-B17122392277-KY438 | 11.2 | 4.72 | 84.2992 | 1 | NEVER | PR | DCB | ORR | 1 | 76 | LUAD | NA | 2 | 2 | Nivolumab |
| F17120989186-KY438-VS-D180312105527-KY438 | 2.48 | 0.87 | 9.82279 | 0.92 | NEVER | PD | NDB | NOR | 1 | 15 | LUAD | NA | 1 | 5 | Nivolumab |
| F17120989191-KY438-VS-B17121390134-KY438 | 9.39 | 1.62 | 61.2669 | 0.16 | EVER | PR | DCB | ORR | 1 | 226 | LUAD | NA | 3 | 2 | Nivolumab |
| F17120989196-KY438-VS-B17120989366-KY438 | 1.03 | 0.41 | 16.4566 | 0 | EVER | PD | NDB | NOR | 1 | 53 | LELC | IV | 1 | 2 | SHR1210 |
| F17120989197-KY438-VS-B17120989368-KY438 | 4.84 | 2.51 | 72.2657 | 0.9 | NEVER | PD | NDB | NOR | 1 | 51 | LUSC | IV | 1 | 3 | SHR1210 |
| F17120989200-KY438-VS-B17121390123-KY438 | 6.93 | 3.53 | 72.1206 | 0.04 | EVER | SD | DCB | NOR | 1 | 378 | LUAD | NA | 1 | 2 | Nivolumab |
| F17120989201-KY438-VS-B17120989370-KY438 | 12.2 | 6.3 | 8.16908 | 0.09 | EVER | SD | NDB | NOR | 1 | 160 | LUSC | IV | 2 | 2 | SHR1210 |
| F17120989205-KY438-VS-B17121390124-KY438 | 18.03 | 8.19 | 386.162 | 0.2 | EVER | PR | DCB | ORR | 0 | 889 | LUAD | IV | 5 | 2 | Nivolumab |
| F17120989208-KY438-VS-B17122392275-KY438 | 1.19 | 0.67 | 10.7873 | 0.49 | NEVER | PD | NDB | NOR | 1 | 38 | LELC | NA | 1 | 2 | Nivolumab |
| F17120989211-KY438-VS-B17120989378-KY438 | 2.42 | 1.19 | 10.3707 | 0.92 | NEVER | PD | NDB | NOR | 1 | 26 | LELC | IV | 3 | 2 | SHR1210 |
| F17120989212-KY438-VS-B17121390125-KY438 | 3.05 | 1.45 | 27.1863 | 0.98 | NEVER | SD | NDB | NOR | 1 | 91 | LUAD | IV | 3 | 2 | Nivolumab |
| F17120989215-KY438-VS-B17120989381-KY438 | 1.61 | 0.74 | 39.3715 | 0.24 | NEVER | PD | NDB | NOR | 1 | 42 | LUAD | NA | 3 | 3 | Nivolumab |
| F17120989224-KY438-VS-B17120989393-KY438 | 0.63 | 0.26 | 5.66314 | 0.97 | EVER | PR | DCB | ORR | 1 | 194 | LUSC | IV | 3 | 2 | Pembro |
| F17120989226-KY438-VS-B17120989398-KY438 | 4.25 | 2.19 | 75.5677 | 0.94 | NEVER | PD | NDB | NOR | 1 | 18 | LUAD | IV | 4 | 2 | Pembro |
| F17120989227-KY438-VS-B17120989399-KY438 | 1.85 | 0.94 | 15.7659 | 0.94 | NEVER | SD | NDB | NOR | 1 | 63 | LELC | IV | 2 | 3 | Pembro |
| F17120989228-KY438-VS-B17120989401-KY438 | 1.87 | 1.09 | 2.6375 | 0.87 | NEVER | PD | NDB | NOR | 1 | 61 | LUAD | IV | 5 | 3 | Pembro |
| F17120989229-KY438-VS-B17120989402-KY438 | 1.45 | 0.73 | 30.6976 | 0.42 | NEVER | PR | DCB | ORR | 1 | 247 | LUAD | IV | 2 | 2 | SHR1210 |
| F17120989243-KY438-VS-B17122392295-KY438 | 3.78 | 2.19 | 153.484 | 0.39 | EVER | SD | NDB | NOR | 1 | 161 | LUSC | IV | 1 | 3 | SHR1210 |
| F17120989250-KY438-VS-B17122392287-KY438 | 2.53 | 1.08 | 13.4381 | 0.99 | NEVER | PD | NDB | NOR | 1 | 34 | LUAD | IV | 3 | 5 | SHR1210 |
| F17120989256-KY438-VS-B17120989428-KY438 | 6.44 | 3.12 | 68.3799 | 0.96 | NEVER | CR | DCB | ORR | 1 | 673 | LUSC | IV | 2 | 4 | SHR1210 |
| F17120989257-KY438-VS-B17120989429-KY438 | 2.8 | 1.88 | 1.15093 | 0.87 | NEVER | SD | NDB | NOR | 1 | 130 | LUAD | IV | 1 | 3 | Pembro |
| F17120989258-KY438-VS-B17120989430-KY438 | 7.25 | 4.52 | 162.18 | 0.48 | NEVER | SD | NDB | NOR | 1 | 110 | LUSC | IV | 2 | 3 | Nivolumab |
| F17120989267-KY438-VS-B17120989441-KY438 | 5.14 | 2.03 | 53.7971 | 1 | EVER | PD | NDB | NOR | 1 | 60 | LUSC | IIIB | 0 | 3 | Pembro |
| F17120989268-KY438-VS-B17120989442-KY438 | 3 | 1.06 | 18.8936 | 0.84 | NEVER | PD | NDB | NOR | 1 | 51 | LUAD | IV | 4 | 4 | SHR1210 |
| F17120989270-KY438-VS-B17120989445-KY438 | 3.25 | 1.22 | 55.5007 | 0.95 | EVER | PD | NDB | NOR | 1 | 60 | LUSC | IV | 3 | 3 | Pembro |
| F17120989277-KY438-VS-B17120989452-KY438 | 0.42 | 0.26 | 8.13808 | 0.98 | EVER | PD | NDB | NOR | 1 | 63 | LUAD | IV | 2 | 4 | Pembro |
| F17120989280-KY438-VS-B17120989458-KY438 | 2.37 | 0.88 | 18.6145 | 0.93 | NEVER | PD | NDB | NOR | 1 | 54 | LUAD | IV | 3 | 2 | Pembro |
| F17120989282-KY438-VS-B17120989460-KY438 | 0.36 | 0.23 | 0 | 0.9 | EVER | PD | NDB | NOR | 1 | 32 | LUAD | IV | 6 | 3 | Pembro |
| F17120989286-KY438-VS-D180312105535-KY438 | 9.74 | 6.17 | 9.02187 | 0.17 | EVER | PD | NDB | NOR | 1 | 60 | LUSC | IV | 1 | 2 | Pembro |
| F17120989294-KY438-VS-D180312105536-KY438 | 0.55 | 0.16 | 1.43983 | 0.98 | EVER | SD | NDB | NOR | 1 | 63 | LUSC | IV | 2 | 4 | ATEZO |
| F17120989295-KY438-VS-D180312105525-KY438 | 1.38 | 0.39 | 3.94907 | 0.99 | EVER | SD | NDB | NOR | 1 | 125 | LUSC | IV | 3 | 3 | ATEZO |
| F17120989306-KY438-VS-B17120989482-KY438 | 1.14 | 0.03 | 0.06444 | 0 | EVER | PR | DCB | ORR | 0 | 466 | LUSC | IV | 2 | 1 | Pembro |
| F17120989317-KY438-VS-B17120989494-KY438 | 9.33 | 3.33 | 62.6212 | 0.06 | EVER | PR | DCB | ORR | 1 | 372 | LUAD | IV | 2 | 2 | Pembro |
| F17120989318-KY438-VS-B17120989433-KY438 | 2 | 0.73 | 44.9135 | 0.85 | NEVER | PD | NDB | NOR | 1 | 40 | LUSC | NA | 2 | 3 | ATEZO |
| T17121390162-KY438-VS-B17120989472-KY438 | 2.82 | 1.05 | 70.0797 | 0.33 | EVER | SD | DCB | NOR | 1 | 252 | LUSC | NA | 1 | 2 | Pembro |
| T17121390164-KY438-VS-D180312105533-KY438 | 1.17 | 0.73 | 4.13497 | 0.54 | NEVER | PD | NDB | NOR | 1 | 63 | LELC | IV | 2 | 2 | Pembro |
| T17121390175-KY438-VS-B17120989466-KY438 | 1.07 | 0.51 | 21.0791 | 0.25 | NEVER | SD | DCB | NOR | 1 | 401 | LUAD | IV | 2 | 1 | Pembro |
| T17121390176-KY438-VS-B17120989465-KY438 | 3.43 | 2.16 | 0 | 0.97 | EVER | PD | NDB | NOR | 1 | 50 | LUAD | IV | 2 | 2 | Pembro |
| T17121390183-KY438-VS-B17120989454-KY438 | 1.55 | 0.88 | 57.5686 | 0.92 | NEVER | SD | NDB | NOR | 1 | 129 | LUAD | IV | 1 | 6 | Pembro |
| T17121390184-KY438-VS-B17120989457-KY438 | 7.6 | 4.32 | 325.873 | 0.06 | EVER | PR | DCB | ORR | 1 | 127 | LUAD | IV | 2 | 3 | Pembro |
| T17121390187-KY438-VS-B17120989461-KY438 | 8.76 | 1.74 | 65.405 | 0.07 | EVER | PD | NDB | NOR | 1 | 51 | LUSC | IV | 1 | 2 | SHR1210 |
| T17121390189-KY438-VS-B17120989463-KY438 | 14.9 | 9 | 17.2015 | 0.13 | EVER | SD | DCB | NOR | 1 | 189 | LUSC | IV | 2 | 5 | Pembro |
| T17121390193-KY438-VS-B17120989450-KY438 | 9.35 | 4.53 | 264.595 | 0.38 | EVER | PR | DCB | ORR | 0 | 637 | LUAD | IV | 1 | 6 | Pembro |
| T17121390195-KY438-VS-B17120989448-KY438 | 9.37 | 5.11 | 16.5274 | 0.08 | EVER | PD | NDB | NOR | 1 | 63 | LUSC | IV | 2 | 3 | Pembro |
| T17121390197-KY438-VS-B17120989436-KY438 | 1.09 | 0.68 | 4.12713 | 0.9 | NEVER | PD | NDB | NOR | 1 | 57 | LUAD | IV | 2 | 4 | SHR1210 |
| T17121390198-KY438-VS-B17120989440-KY438 | 7.82 | 4.42 | 0 | 0 | EVER | PD | NDB | NOR | 1 | 50 | LUAD | IV | 5 | 2 | SHR1210 |
| T17121390202-KY438-VS-B17120989490-KY438 | 8.54 | 4.37 | 248.328 | 0.12 | EVER | SD | NDB | NOR | 1 | 130 | LUAD | IV | 1 | 4 | Pembro |
| T17121390203-KY438-VS-B17120989493-KY438 | 10.99 | 6.55 | 0.78566 | 0.01 | EVER | PD | NDB | NOR | 1 | 64 | ASC | IV | 2 | 5 | Pembro |
| T17121390205-KY438-VS-B17120989435-KY438 | 3.87 | 0.86 | 42.5121 | 0.2 | NEVER | PD | NDB | NOR | 1 | 65 | LUAD | IV | 1 | 2 | Pembro |
| T17121390207-KY438-VS-B17120989417-KY438 | 4.74 | 2.68 | 138.948 | 0.19 | NEVER | PD | NDB | NOR | 1 | 33 | LUAD | IV | 5 | 6 | SHR1210 |
| T17121390211-KY438-VS-B17120989407-KY438 | 1.97 | 0.86 | 52.234 | 0.31 | NEVER | PD | NDB | NOR | 1 | 63 | LUAD | IV | 3 | 3 | Pembro |
| T17121390212-KY438-VS-B17120989412-KY438 | 2.34 | 0.66 | 4.28143 | 0 | EVER | PR | DCB | ORR | 1 | 315 | LUAD | IV | 1 | 6 | Pembro |
| T17121390214-KY438-VS-B17120989394-KY438 | 0.57 | 0.22 | 6.12188 | 0 | NEVER | PD | NDB | NOR | 1 | 56 | LUSC | IV | 2 | 2 | SHR1210 |
| T17121390220-KY438-VS-B17120989425-KY438 | 8.79 | 4.49 | 264.966 | 0.2 | EVER | SD | NDB | NOR | 0 | 126 | LUSC | IV | 2 | 3 | SHR1210 |
| T17121390227-KY438-VS-B17120989414-KY438 | 5.49 | 2.81 | 226.571 | 0.02 | EVER | PR | DCB | ORR | 1 | 674 | LUAD | IV | 3 | 6 | SHR1210 |
| T17121390228-KY438-VS-B17120989437-KY438 | 0.54 | 0.35 | 3.03344 | 0 | NEVER | PR | DCB | ORR | 1 | 332 | LUAD | IV | 3 | 2 | SHR1210 |
| T17121390229-KY438-VS-B17120989438-KY438 | 0.29 | 0.19 | 0 | 0 | NEVER | PD | NDB | NOR | 1 | 40 | LUAD | IV | 3 | 3 | Pembro |
| T17121390230-KY438-VS-B17120989456-KY438 | 0.6 | 0.29 | 0 | 0.97 | NEVER | SD | NDB | NOR | 1 | 174 | LUAD | IV | 5 | 3 | SHR1210 |
| T17121390231-KY438-VS-B17121390128-KY438 | 0.9 | 0.1 | 4.07932 | 0.63 | NEVER | PD | NDB | NOR | 1 | 55 | LUAD | IV | 3 | 2 | Pembro |
| T17121390232-KY438-VS-B17121390137-KY438 | 14.21 | 8.64 | 648.134 | 0.01 | NEVER | SD | NDB | NOR | 1 | 108 | LUSC | IV | 1 | 2 | Pembro |
| T17121390235-KY438-VS-B17121390140-KY438 | 1.14 | 0.41 | 12.3056 | 0.57 | NEVER | PD | NDB | NOR | 1 | 35 | LUAD | IV | 2 | 2 | Pembro |
| T17121390239-KY438-VS-D180312105532-KY438 | 1.99 | 0.38 | 3.44699 | 0 | EVER | PD | NDB | NOR | 1 | 65 | LUAD | IV | 4 | 2 | Pembro |
| T17121390240-KY438-VS-D180312105537-KY438 | 7.44 | 2.48 | 146.741 | 0.12 | EVER | PR | DCB | ORR | 0 | 503 | LUSC | IV | 1 | 1 | Pembro |

| **Supplementary Table 4. Summary of ioTNL and clinical characteristics in NPC cohort.** | | | | | | | | | | | | | |
| --- | --- | --- | --- | --- | --- | --- | --- | --- | --- | --- | --- | --- | --- |
| **Sample** | **TMB** | **TNL** | **ioTNL** | **Age** | **Gender** | **Smoke** | **FarMetastasis** | **Drug** | **stat** | **PFS** | **Benefit** | **ORR** | **DCB** |
| F17120989190-KY438-VS-B17120989361-KY438 | 6.04 | 2.34 | 51.5622 | 35 | Male | NEVER | 2 | SHR1210 | 1 | 43 | PD | NOR | NDB |
| F17120989194-KY438-VS-B17120989363-KY438 | 2.78 | 1.21 | 10.3808 | 56 | Male | EVER | 1 | SHR1210 | 1 | 162 | SD | NOR | NDB |
| F17120989195-KY438-VS-B17120989365-KY438 | 4.47 | 0.95 | 7.1694 | 51 | Male | EVER | 3 | SHR1210 | 1 | 108 | SD | NOR | NDB |
| F17120989198-KY438-VS-B17120989369-KY438 | 4.08 | 1.24 | 24.9171 | 37 | Male | NEVER | 2 | SHR1210 | 1 | 245 | PR | ORR | DCB |
| F17120989202-KY438-VS-B17120989371-KY438 | 1.69 | 0.78 | 2.64078 | 42 | Female | NEVER | 2 | SHR1210 | 1 | 50 | PD | NOR | NDB |
| F17120989203-KY438-VS-B17120989372-KY438 | 2.3 | 0.2 | 2.37269 | 61 | Male | EVER | 3 | SHR1210 | 1 | 53 | PD | NOR | NDB |
| F17120989204-KY438-VS-B17120989373-KY438 | 2.27 | 1.22 | 8.68228 | 48 | Male | NEVER | 2 | SHR1210 | 1 | 52 | PD | NOR | NDB |
| F17120989209-KY438-VS-B17120989376-KY438 | 4.1 | 2.02 | 55.0944 | 33 | Female | NEVER | 3 | SHR1210 | 1 | 31 | PD | NOR | NDB |
| F17120989210-KY438-VS-B17120989377-KY438 | 2.78 | 1.67 | 43.4561 | 55 | Male | NEVER | 1 | Nivolumab | 1 | 500 | PR | ORR | DCB |
| F17120989213-KY438-VS-B17120989379-KY438 | 15.94 | 6.04 | 50.6097 | 56 | Male | NEVER | 1 | Nivolumab | 1 | 105 | SD | NOR | NDB |
| F17120989214-KY438-VS-B17120989380-KY438 | 3.85 | 2.58 | 24.5605 | 25 | Male | NEVER | 1 | SHR1210 | 1 | 217 | PR | ORR | DCB |
| F17120989216-KY438-VS-B17120989382-KY438 | 2.83 | 1.28 | 39.856 | 61 | Male | EVER | 1 | Nivolumab | 1 | 25 | PD | NOR | NDB |
| F17120989217-KY438-VS-B17120989383-KY438 | 3.33 | 1.41 | 28.7436 | 29 | Male | NEVER | 1 | Nivolumab | 1 | 54 | PD | NOR | NDB |
| F17120989218-KY438-VS-B17120989384-KY438 | 5.68 | 2.36 | 77.0196 | 36 | Female | NEVER | 3 | SHR1210 | 1 | 50 | PD | NOR | NDB |
| F17120989219-KY438-VS-B17120989385-KY438 | 2.77 | 1.78 | 57.7457 | 41 | Male | NEVER | 3 | SHR1210 | 1 | 52 | PD | NOR | NDB |
| F17120989222-KY438-VS-B17120989390-KY438 | 2.56 | 0.29 | 1.78514 | 38 | Male | NEVER | 1 | SHR1210 | 1 | 52 | PD | NOR | NDB |
| F17120989225-KY438-VS-B17120989397-KY438 | 2.34 | 1.04 | 38.8215 | 61 | Male | EVER | 2 | SHR1210 | 1 | 245 | SD | NOR | DCB |
| T17121390199-KY438-VS-B17120989408-KY438 | 1.37 | 0.13 | 1.3395 | 56 | Male | EVER | 2 | SHR1210 | 1 | 50 | PD | NOR | NDB |
| T17121390209-KY438-VS-B17120989410-KY438 | 1.34 | 0.51 | 21.1782 | 59 | Male | NEVER | 2 | Nivolumab | 1 | 105 | SD | NOR | NDB |
| T17121390224-KY438-VS-B17120989413-KY438 | 1.58 | 0.98 | 47.8589 | 60 | Male | NEVER | 3 | Nivolumab | 1 | 49 | PD | NOR | NDB |
| T17121390206-KY438-VS-B17120989415-KY438 | 1.99 | 1.09 | 29.0791 | 59 | Female | NEVER | 3 | Nivolumab | 1 | 103 | SD | NOR | NDB |
| T17121390226-KY438-VS-B17120989416-KY438 | 2.68 | 1.29 | 83.1408 | 39 | Female | NEVER | 0 | Nivolumab | 1 | 158 | SD | NOR | NDB |
| T17121390204-KY438-VS-B17120989418-KY438 | 2.47 | 0.85 | 26.1296 | 53 | Female | NEVER | 2 | Nivolumab | 1 | 476 | PR | ORR | DCB |
| F17120989247-KY438-VS-B17120989420-KY438 | 1.94 | 0.92 | 6.17803 | 61 | Male | NEVER | 1 | Nivolumab | 1 | 52 | SD | NOR | NDB |
| T17121390215-KY438-VS-B17120989421-KY438 | 2.07 | 0.89 | 31.83 | 34 | Male | EVER | 3 | SHR1210 | 1 | 52 | PD | NOR | NDB |
| T17121390219-KY438-VS-B17120989422-KY438 | 3.29 | 1.44 | 99.3122 | 43 | Male | NEVER | 3 | Nivolumab | 1 | 17 | PD | NOR | NDB |
| F17120989253-KY438-VS-B17120989424-KY438 | 2.95 | 0.03 | 0.66951 | 47 | Male | NEVER | 2 | Nivolumab | 1 | 389 | SD | NOR | DCB |
| T17121390201-KY438-VS-B17120989431-KY438 | 1.4 | 0.41 | 15.1387 | 38 | Female | NEVER | 3 | SHR1210 | 1 | 152 | SD | NOR | NDB |
| F17120989271-KY438-VS-B17120989446-KY438 | 4.09 | 1.56 | 23.1438 | 41 | Male | EVER | 3 | Nivolumab | 1 | 48 | PD | NOR | NDB |
| F17120989281-KY438-VS-B17120989459-KY438 | 7.3 | 2.8 | 104.629 | 45 | Male | EVER | 3 | SHR1210 | 1 | 50 | PD | NOR | NDB |
| T17121390177-KY438-VS-B17120989464-KY438 | 2.75 | 1.55 | 64.1432 | 47 | Male | NEVER | 2 | SHR1210 | 1 | 164 | SD | NOR | NDB |
| T17121390180-KY438-VS-B17120989467-KY438 | 1.07 | 0.69 | 16.2119 | 48 | Male | NEVER | 3 | SHR1210 | 1 | 50 | PD | NOR | NDB |
| T17121390191-KY438-VS-B17120989468-KY438 | 2.51 | 1.59 | 0 | 42 | Male | NEVER | 2 | SHR1210 | 1 | 98 | SD | NOR | NDB |
| T17121390192-KY438-VS-B17120989469-KY438 | 1.94 | 0.48 | 1.28162 | 53 | Male | NEVER | 4 | SHR1210 | 1 | 50 | PD | NOR | NDB |
| F17120989290-KY438-VS-B17120989471-KY438 | 3.36 | 1.61 | 27.3798 | 66 | Male | EVER | 1 | SHR1210 | 1 | 108 | PR | ORR | DCB |
| T17121390165-KY438-VS-B17120989473-KY438 | 2.62 | 1.85 | 79.6843 | 47 | Male | NEVER | 1 | SHR1210 | 0 | 580 | PR | ORR | DCB |
| T17121390166-KY438-VS-B17120989475-KY438 | 1.64 | 1.01 | 33.9072 | 32 | Male | NEVER | 2 | SHR1210 | 1 | 250 | PR | ORR | DCB |
| F17120989297-KY438-VS-B17120989476-KY438 | 1.42 | 0.68 | 6.26299 | 39 | Male | NEVER | 2 | SHR1210 | 1 | 50 | PD | NOR | NDB |
| T17121390163-KY438-VS-B17120989477-KY438 | 1.78 | 0.76 | 41.6698 | 54 | Male | NEVER | 2 | SHR1210 | 1 | 51 | PD | NOR | NDB |
| T17121390172-KY438-VS-B17120989478-KY438 | 1.32 | 0.63 | 2.66012 | 59 | Male | NEVER | 2 | SHR1210 | 1 | 23 | PD | NOR | NDB |
| F17120989300-KY438-VS-B17120989479-KY438 | 2.97 | 1.8 | 40.5309 | 28 | Male | NEVER | 1 | SHR1210 | 1 | 50 | PD | NOR | NDB |
| T17121390168-KY438-VS-B17120989481-KY438 | 2.31 | 1.26 | 59.4841 | 52 | Male | EVER | 1 | SHR1210 | 1 | 52 | PD | NOR | NDB |
| T17121390181-KY438-VS-B17120989483-KY438 | 3.69 | 2.05 | 93.6559 | 64 | Male | NEVER | 2 | SHR1210 | 1 | 53 | PD | NOR | NDB |
| T17121390182-KY438-VS-B17120989484-KY438 | 1.99 | 0.57 | 10.2068 | 51 | Male | NEVER | 2 | SHR1210 | 1 | 56 | PD | NOR | NDB |
| F17120989310-KY438-VS-B17120989485-KY438 | 3.89 | 2.01 | 45.2421 | 72 | Male | EVER | 1 | Nivolumab | 1 | 166 | SD | NOR | NDB |
| F17120989311-KY438-VS-B17120989486-KY438 | 1.89 | 0.57 | 18.9128 | 60 | Female | NEVER | 2 | Nivolumab | 1 | 40 | PD | NOR | NDB |
| T17121390223-KY438-VS-B17120989499-KY438 | 1.46 | 0.54 | 6.60499 | 36 | Male | NEVER | 2 | SHR1210 | 0 | 430 | PR | ORR | DCB |
| T17121390221-KY438-VS-B17120989500-KY438 | 2.63 | 1.52 | 58.8314 | 56 | Male | EVER | 2 | SHR1210 | 0 | 584 | PR | ORR | DCB |
| T17121390170-KY438-VS-B17120989504-KY438 | 3.2 | 1.42 | 104.118 | 57 | Male | NEVER | 1 | SHR1210 | 0 | 117 | PR | ORR | DCB |
| T17121390222-KY438-VS-B17120989505-KY438 | 2.02 | 0.95 | 43.9688 | 48 | Male | EVER | 2 | SHR1210 | 0 | 396 | PR | ORR | DCB |
| T17121390160-KY438-VS-B17120989506-KY438 | 2.01 | 0.89 | 39.799 | 32 | Male | NEVER | 1 | SHR1210 | 1 | 50 | PD | NOR | NDB |
| T17121390159-KY438-VS-B17120989507-KY438 | 0.22 | 0.06 | 0 | 23 | Female | NEVER | 3 | SHR1210 | 1 | 50 | PD | NOR | NDB |
| T17121390161-KY438-VS-B17120989508-KY438 | 2.35 | 1.62 | 34.706 | 69 | Female | NEVER | 3 | SHR1210 | 0 | 609 | PR | ORR | DCB |
| F17120989322-KY438-VS-B17121390135-KY438 | 3.24 | 1.37 | 37.1151 | 42 | Male | NEVER | 0 | IPI | 1 | 33 | PD | NOR | NDB |
| T17121390233-KY438-VS-B17121390138-KY438 | 3.48 | 1.39 | 73.2447 | 28 | Male | NEVER | 1 | SHR1210 | 1 | 250 | PR | ORR | DCB |
| T17121390234-KY438-VS-B17121390139-KY438 | 4.46 | 1.98 | 75.3611 | 40 | Male | EVER | 0 | IPI | 1 | 19 | PD | NOR | NDB |
| F17120989232-KY438-VS-B17122392291-KY438 | 3.64 | 1.48 | 16.7915 | 40 | Male | EVER | 1 | Nivolumab | 1 | 107 | SD | NOR | NDB |
| T17121390213-KY438-VS-B17122392305-KY438 | 1.93 | 0.73 | 26.0237 | 45 | Female | NEVER | 1 | Nivolumab | 0 | 748 | PR | ORR | DCB |
| F17120989323-KY438-VS-B17122392307-KY438 | 2.3 | 0.85 | 11.4598 | 48 | Female | NEVER | 0 | IPI | 1 | 58 | PD | NOR | NDB |
| T17121390167-KY438-VS-D180312105528-KY438 | 1.07 | 0.47 | 24.4358 | 43 | Male | NEVER | 1 | SHR1210 | 1 | 108 | SD | NOR | NDB |
| F17120989307-KY438-VS-D180312105540-KY438 | 2.27 | 0.94 | 26.4042 | 47 | Male | NEVER | 1 | SHR1210 | 1 | 333 | PR | ORR | DCB |
